# Species Diversity of *Amanita* Section *Vaginatae* in Eastern China, with a Description of Four New Species

**DOI:** 10.3390/jof9080862

**Published:** 2023-08-19

**Authors:** Yang-Yang Cui, Yan-Jia Hao, Ting Guo, Zhu L. Yang, Qing Cai

**Affiliations:** 1 Key Laboratory for Plant Diversity and Biogeography of East Asia, Kunming Institute of Botany, Chinese Academy of Sciences, Kunming 650201, China; cuiyangyang@mail.kib.ac.cn; 2 Yunnan Key Laboratory for Fungal Diversity and Green Development, Kunming 650201, China; 3 College of Horticulture, Anhui Agricultural University, Hefei 230036, China; 4 Institute of Edible Fungi, Shanghai Academy of Agricultural Sciences, Shanghai 201403, China; 5 Key Laboratory of Edible Fungal Resources and Utilization (South), Ministry of Agriculture and Rural Affairs, Shanghai 201403, China; 6 National Engineering Research Center of Edible Fungi, Shanghai 201403, China

**Keywords:** *Agaricales*, ectomycorrhizal fungi, Funga, morphology, integrative taxonomy

## Abstract

Species of *Amanita* sect. *Vaginatae* (Fr.) Quél. are challenging to delimitate due to the morphological similarity or morphostasis among different taxa. In this study, a multi-locus (nuc rDNA region encompassing the internal transcribed spacers 1 and 2 with the 5.8S rDNA, the D1–D3 domains of nuc 28S rDNA, partial sequences of translation elongation factor 1-a, and the second largest subunit of RNA polymerase II) phylogeny was employed to investigate the species diversity of the section in eastern China. Sixteen species were recognized, including four new species; namely, *A. circulata*, *A. multicingulata*, *A. orientalis*, and *A. sinofulva*. They were documented with illustrated descriptions, ecological evidence, and comparisons with similar species. A key to the species of the section from eastern China is provided.

## 1. Introduction

*Amanita* Pers. is a cosmopolitan genus with about 700 accepted species [1,2,3,4,5,6,7,8,9,10,11,12,13]. According to the most recent comprehensive phylogenetic studies, *Amanita* is divided into three subgenera and 11 sections [2]. In these sections, *A*. sect. *Vaginatae* (Fr.) Quél. is the most species-rich (http://www.amanitaceae.org/; accessed on 1 January 2023). The section is characterized by a striate and non-appendiculate pileus, a bulbless stipe base with saccate volva or warts arranged in incomplete belts, mostly absent annulus, inamyloid basidiospores, and an absence of clamps.

Species of *A.* sect. *Vaginatae* are distributed worldwide and can form ectomycorrhizal associations with plants of more than ten families, such as Fagaceae, Pinaceae, and Dipterocarpaceae [1,14,15,16,17,18]. It is challenging to delimitate and recognize the species of the section due to the morphological similarity or morphostasis among different taxa. As currently circumscribed, ca. 145 species have been described and accepted [2,4,5,14,19,20,21,22,23,24,25,26,27,28,29,30,31,32,33,34] (http://www.amanitaceae.org/; accessed on 1 January 2023). Asia and Europe are the most species-rich areas of the section, in which 48 and 47 taxa have been reported, respectively. Thirty and five taxa have been described from North and South America, respectively. In Africa and Oceania, seventeen species have been described (http://www.amanitaceae.org/; accessed on 1 January 2023).

In China, 33 taxa of *A.* sect. *Vaginatae* have been reported [1,2,21,35]. Twenty-seven of them can be found in southwestern China. Southern and eastern China are the second and third most species-rich areas, with six and five species. Three species have been reported in central and northeastern China, respectively. The majority of them are distributed in more than one area. There are nine endemic species in southwestern China. Only one species is restricted in the southern, central, and northeastern parts, respectively. The five taxa in eastern China—namely *A. cingulata* J.W. Liu and Zhu L. Yang, *A. hamadae* Nagas. and Hongo, *A. olivaceofusca* Y.Y. Cui et al., *A. ovalispora* Boedijn, and *A. pallidozonata* Y.Y. Cui et al.—also occur in central, southern, and southwestern China [2].

During our investigations of macrofungi in eastern China, numerous specimens of *A.* sect. *Vaginatae* were collected. In this study, we applied integrative taxonomy, including morphological characters, multi-locus phylogenetic evidence, and ecological data, to elucidate the species diversity of *A.* sect. *Vaginatae* in eastern China. A key to all of the species found in the area is provided.

## 2. Materials and Methods

### 2.1. Taxon Sampling

Sixty-one specimens of *A.* sect. *Vaginatae* were examined. Among them, 50 were collected from eastern China, and the rest were from southwestern China. For each collection, a part of the basidioma was dried with silica gel for DNA extraction. The remaining materials were then dried at 45–50 °C with an electronic food dehydrator. At the same time, the specimen information, host trees, altitudes, locations, collectors, and dates were recorded, and photos of the fruiting bodies were taken. The location information and ecological habits of the specimens mentioned above are stated in the results section. All specimens examined in this work were deposited in the Herbarium of the Kunming Institute of Botany, Chinese Academy of Sciences (KUN-HKAS), and the Edible-medicinal Fungal Herbarium of Anhui Agriculture University (EFHAAU).

### 2.2. Morphological Observation

The macroscopic descriptions are based on detailed field notes and photographs of fresh basidiomata. Color codes indicated in the descriptions are from Kornerup and Wanscher [36]. Microscopic features were studied with light microscopy using dried material rehydrated in 5% KOH and, when necessary, dyed with Congo Red. Melzer’s reagent was used to check the amyloidity of basidiospores. In the description of the basidiospores, the abbreviation (n/m/p) represents n basidiospores measured from m basidiomata of p collections. Dimensions for the basidiospores are given using a range notation of the form (a–) b–c (–d). The range b–c contains a minimum of 90% of the measured values. Extreme values, a or d, are given in parentheses. Q represents the ‘length/width ratio’ of a basidiospore in the side view. Qm means the average Q of all basidiospores measured ± sample standard deviation.

### 2.3. DNA Extraction, PCR Amplification, and Sequencing

Genomic DNA was extracted from silica gel-dried material or herbarium specimens using the modified CTAB method [37]. The primer pairs LR0R/LR5 [38], ITS1F/ITS4 [39], 983F/1567R [40], and Am-6F/Am-7R [41] were used to amplify the large subunit D1–D3 domains of nuc 28S rDNA (nrLSU), nuc rDNA internal transcribed spacer ITS1-5.8S-ITS2 (ITS), partial sequences of translation elongation factor 1-a (*tef1-α*), and the second largest subunit of RNA polymerase II (*rpb2*), respectively. Protocols for the polymerase chain reactions (PCR) and sequencing followed those in Cai et al. [41] and the reference therein. All sequences analyzed in this study were deposited at GenBank and are listed in Table 1.

### 2.4. Sequence Alignments and Phylogenetic Analyses

According to the most recent comprehensive multi-locus phylogenetic analyses of *A*. sect. *Vaginatae*, the ITS sequences were not included [33]. Therefore, two datasets, the multi-locus dataset (nrLSU, *tef1-α* and *rpb2*) and the ITS sequences matrix, were compiled to infer the phylogeny of the section, respectively. For the ITS dataset, sequences of the new taxa were initially blasted in GenBank. The most closely related sequences (nucleotide identities >90%) were retrieved to complement the ITS matrix with one to two representatives per species. For the combined dataset, all known species of the section with sequences of the gene fragments employed in this study were included [2,4,5,21,22,33]. According to recent phylogenetic analyses, *A. muscaria* (L.) Lam., *A. parvipantherina* Zhu L. Yang et al., *A. caesarea* (Scop.) Pers., and *A. yuaniana* Zhu L. Yang of *A*. sect. *Amanita* and sect. *Caesareae* Singer ex Singer were selected as outgroups [2]. Sequences of all gene fragments were separately aligned with MAFFT 7 [42] and manually optimized in BioEdit 7 [43]. For the two datasets, the introns of *tef1-α* and *rpb2* were excluded because of the difficulty in alignment. The ambiguously aligned regions of nrLSU and ITS were eliminated using Gblocks 0.91b [44] with the “less stringent selection” parameter set. The final alignments of both datasets were deposited in TreeBASE (30635).

Single-gene analyses were carried out for the concatenated matrix to detect possible incongruence among individual genes based on the Maximum Likelihood (ML) method. Because no well-supported bootstrap value (BS > 70%) was detected (Figures S1–S3), the resulting alignments of nrLSU, *tef1-α*, and *rpb2* were concatenated using Geneious v9.1.3 [45]. The best partition schemes and evolutionary models of the two datasets were selected using PartitionFinder V2.1.1 [46]. ITS was divided into three blocks: ITS1, ITS2, and 5.8S. The concatenated matrix was partitioned according to gene fragments and codon positions, including nrLSU, *tef1-α_*codon1, *tef1-α_*codon2, *tef1-α_*codon3, *rpb2_*codon1, *rpb2_*codon2, and *rpb2_*codon3. Thus, three and seven blocks were predefined for the ITS and combined datasets, respectively. The two datasets were then analyzed using RAxML v8.2.4 [47] and MrBayes v3.2.6 [48] for ML and Bayesian Inference (BI) analyses, respectively. In the ML analyses, the statistical supports were obtained using rapid bootstrapping with 1000 replicates, and the other parameters used the default settings. Some of the selected models could not be implemented in RAxML; thus, the GTR + I + G model, which included all of the parameters of the selected model, was used for all partitioned data. For BI analyses, four Markov Chain Monte Carlo (MCMC) chains were run simultaneously for 5 million generations under the best partition schemes and evolutionary models selected using PartitionFinder, and trees were sampled every 1000 generations. Runs were automatically terminated when the average standard deviation of split frequencies fell below 0.01 and the ESS values exceeded 200 [48]. Tracer v1.5 (http://tree.bio.ed.ac.uk/software/tracer/, accessed on 20 January 2023) was used to confirm the chain convergence. Subsequently, the sampled trees were summarized, and posterior probabilities were obtained by discarding the first 25% of generations as burn-in.

**Table 1:** Information on specimens used in multi-locus phylogenetic analyses and their GenBank accession numbers. Sequences newly generated in this study are indicated in bold.

Species	Voucher	Locality	GenBank No.			Reference
			nrLSU	*tef1-α*	*rpb2*	
*A. albidostipes*	HKAS57358	China	MH486756	MH508983	–	[2]
*A. albidostipes*	HKAS95189	China	MH486757	–	–	[2]
*A. angustilamellata*	HKAS24158	China	AF024440	–	–	[2]
*A*. cf. *angustilamellata*	HKAS83453	China	MH486430	–	–	[2]
*A*. cf. *angustilamellata*	HKAS89451	China	MH486431	MH508716	MH485910	[2]
*A. annulata*	MHKMU L. P. Tang 1671	China	MZ005570	–	–	[21]
*A. basiana*	RET 308-4	Italy	KP258987	–	–	Direct sub.
*A. battarrae*	HKAS92090	China	MH486388	MH508689	MH485880	[2]
*A. battarrae*	MB-000643	Germany	MH486389	MH508690	MH485881	[2]
*A. brunneofuliginea*	HKAS29508	China	AF024442	–	–	[2]
*A. brunneofuliginea*	HKAS89226	China	MH486391	MH508691	MH485883	[2]
*A. brunneoprocera*	BZ2015-24	Thailand	MF461553	–	MF440412	[4]
***A. brunneoprocera***	**EFHAAU3796**	**China**	**OR042681**	**OR046329**	**OR051723**	**This study**
***A. brunneoprocera***	**EFHAAU4162**	**China**	**OR042682**	**OR046330**	**OR051724**	**This study**
*A. brunneoprocera*	HKAS97514	China	MH486390	–	MH485882	[2]
*A. brunneoprocera*	HKAS98435	China	MH486391	MH508691	MH485883	[2]
*A. brunneosquamata*	BZ2015-73	Thailand	MF461563	–	MF440422	[4]
*A. brunneoumbonata*	BZ2015-67	Thailand	MF461561	–	MF440420	[4]
***A. brunneoumbonata***	**EFHAAU131**	**China**	**OR042683**	**OR046370**	**OR051725**	**This study**
‘*A. ceciliae*’	ASIS26247	South Korea	KU139437	–	–	Direct sub.
‘*A. ceciliae*’	ASIS26935	South Korea	KU139439	–	–	Direct sub.
*A. ceciliae*	C. Bas9341	The Netherlands	AF024444	–	–	[49]
‘*A. ceciliae*’	KA12-0758	South Korea	KF021668	–	–	[32]
‘*A. ceciliae*’	KA12-0916	South Korea	KF021669	–	–	[32]
*A. changtuia*	HKAS92100	China	MH486442	MH508724	MH485919	[2]
*A. chiui*	HKAS76328	China	MH486447	MH508727	MH485930	[2]
*A. cinctipes*	HKAS101388	China	MH486448	–	–	[2]
*A. cinctipes*	HKAS78465	China	MH486449	–	–	[2]
*A. cingulata*	HKAS100640	China	MH486454	MH508731	MH485935	[2]
*A. cingulata*	HKAS75600	China	KY949583	–	–	[50]
*A. cinnamomea*	BZ2015-45	Thailand	MF461555	–	MF440414	[4]
*A. cinnamomea*	BZ2015-48	Thailand	MF461557	–	MF440416	[4]
***A. circulata***	**HKAS127629**	**China**	**OR042717**	**OR046356**	**OR051749**	**This study**
***A. circulata***	**HKAS127639**	**China**	**OR042715**	**OR046355**	**OR051747**	**This study**
***A. circulata***	**HKAS101238**	**China**	**OR042718**	**OR046357**	**OR051750**	**This study**
***A. circulata***	**HKAS56815**	**China**	**OR042719**	**–**	**–**	**This study**
***A. circulata***	**HKAS57535**	**China**	**OR042720**	**–**	**–**	**This study**
***A. circulata***	**HKAS57543**	**China**	**OR042721**	**OR046358**	**–**	**This study**
***A. circulata***	**HKAS67955**	**China**	**OR042722**	**OR046359**	**–**	**This study**
***A. circulata***	**HKAS76411**	**China**	**OR042723**	**–**	**–**	**This study**
***A. circulata***	**HKAS97054**	**China**	**OR042724**	**OR046360**	**OR051751**	**This study**
***A. circulata***	**HKAS97543**	**China**	**OR042725**	**OR046361**	**OR051752**	**This study**
***A. circulata***	**HKAS97784**	**China**	**OR042726**	**OR046362**	**OR051753**	**This study**
***A. circulata***	**HKAS128052**	**China**	**OR042716**	**OR046354**	**OR051748**	**This study**
***A*** **. cf. *circulata***	**EFHAAU4143**	**China**	**OR042684**	**–**	**–**	**This study**
***A.*** **cf. *circulata***	**HKAS128051**	**China**	**OR042704**	**OR046331**	**–**	**This study**
*A. cistetorum*	RET 293-5	Italy	MK536604	–	–	Direct sub.
*A. colombiana*	ANDES_F910_NVE410	Colombia	KT008041	KT008012	–	[23]
*A. constricta*	BW_Mycoblitz IV_2	USA	HQ539684	–	–	Direct sub.
*A. cornelii*	CAL 1337	India	KX528072	–	–	Direct sub.
*A. craseoderma*	INPA No. 265158	Brazil	ON392651	ON492138	ON492104	[33]
*A. crebresulcata*	INPA No. 265178	Brazil	ON392663	ON540337	ON492115	[33]
*A.* cf. *daimonioctantes*	TRTC155757	Canada	ON392639	ON492125	–	[33]
*A. emodotrygon*	MFM-219	Pakistan	MF491881	–	–	[5]
*A. emodotrygon*	SUA902	Pakistan	MF491880	–	–	[5]
*A. flammeola*	JD960	DR Congo	–	–	MF440424	[4]
*A. flavidocerea*	BZ2015-59	Thailand	MF461559	–	MF440418	[4]
*A. flavidocerea*	BZ2015-60	Thailand	MF461560	–	MF440419	[4]
*A. flavidogrisea*	BZ2015-44	Thailand	MF461554	–	MF440413	[4]
*A. friabilis*	AF2529	Belgium	–	–	MF440404	[4]
*A. fuligineodisca*	ANDES_F823_NVE324	Colombia	KT008039	KT008011	–	[23]
‘*A. fulva*’	ASIS26398	South Korea	KU139446	–	–	Direct sub.
*A. fulva*	HKAS96168	Austria	MH486555	MH508826	MH486022	[2]
*A. fulva*	N. Arnold2	The Netherlands	AF024455	–	–	[49]
*A.* aff. *fulva*	HKAS29518	China	AF024456	–	–	[49]
*A. glarea*	LAH35044	Pakistan	KY781175	–	–	[19]
***A. griseofolia***	**EFHAAU555**	**China**	**OR042689**	**OR046334**	**OR051728**	**This study**
*A. griseofolia*	HKAS38159	China	AY436488	–	–	[51]
*A. griseofolia*	HKAS54443	China	MH486564	MH508835	MH486029	[2]
*A. griseofusca*	LAH35366	Pakistan	MH241056	MH282854	–	[20]
*A. griseofusca*	SWAT000137	Pakistan	MH241058	–	–	[20]
*A. griseoumbonata*	HKAS92103	China	MH486578	MH508847	MH486040	[2]
***A. hamadae***	**EFHAAU3013**	**China**	**OR042690**	**OR046335**	**OR051729**	**This study**
*A. hamadae*	HKAS79081	China	MH486585	–	MH486047	[2]
*A. hamadae*	HKAS83451	China	MH486586	MH508853	MH486048	[2]
*A. lignitincta*	HKAS29512	China	AF024461	–	–	[49]
*A. lignitincta*	HKAS69411	China	MH486625	–	MH508883	[2]
*A. lippiae*	RET 418-2	Brazil	NG_057062	–	–	Direct sub.
*A. liquii*	HKAS36611	China	AY436493	–	–	[51]
*A. liquii*	HKAS54568	China	JF710794	KU714525	KU714584	[52]
*A. liquii*	HKAS93915	China	MH486628	MH508886	MH486078	[2]
*A. luteoparva*	BZ2015-46	Thailand	MF461556	–	MF440415	[4]
*A. madagascariensis*	JEIC0515	Guinea	ON843338	ON894331	ON854980	[33]
*A. magnivolvata*	AF2528	Belgium	MF461551	–	MF440403	[4]
*A. malleata*	AM91-255	Belgium	–	–	MF440406	[4]
*A. mansehraensis*	LAH31005	Pakistan	MG195982	MH495970	–	[22]
*A. mansehraensis*	LAH31006	Pakistan	MG195983	–	–	[22]
***A. multicingulata***	**HKAS128054**	**China**	**OR042711**	**OR046350**	**OR051743**	**This study**
***A. multicingulata***	**HKAS128049**	**China**	**OR042712**	**OR046351**	**OR051744**	**This study**
***A. multicingulata***	**HKAS127630**	**China**	**OR042713**	**OR046352**	**OR051745**	**This study**
***A. multicingulata***	**HKAS127631**	**China**	**OR042714**	**OR046353**	**OR051746**	**This study**
***A. multicingulata***	**HKAS127632**	**China**	**OR042705**	**OR046346**	**OR051739**	**This study**
***A. multicingulata***	**HKAS127633**	**China**	**OR042706**	**OR046347**	**OR051740**	**This study**
***A. multicingulata***	**HKAS127634**	**China**	**OR042707**	**OR046348**	**OR051741**	**This study**
***A. multicingulata***	**HKAS127635**	**China**	**OR042708**	**OR046349**	**OR051742**	**This study**
***A. multicingulata***	**HKAS127636**	**China**	**OR042709**	**–**	**–**	**This study**
***A. multicingulata***	**HKAS127637**	**China**	**OR042710**	**–**	**–**	**This study**
*A. neocinctipes*	HKAS79627	China	MH486653	MH508910	MH486103	[2]
*A. neocinctipes*	HKAS78463	China	–	–	MH486102	[2]
*A. nivalis*	R. Watling 17489	The Netherlands	AF024466	–	–	[49]
*A. olivaceofusca*	HKAS97581	China	MH486691	–	MH486127	[2]
*A. olivaceofusca*	HKAS80243	China	MH486689	MH508934	MH486125	[2]
*A. olivaceogrisea*	AF2427	The Netherlands	–	–	MF440402	[4]
*A. olivovaginata*	SUA138	Pakistan	MF491875	–	–	Direct sub.
*A. olivovaginata*	SUA939	Pakistan	MF491873	–	–	Direct sub.
***A. orientalis***	**EFHAAU1367**	**China**	**OR042698**	**–**	**OR051735**	**This study**
***A. orientalis***	**HKAS127638**	**China**	**OR042697**	**OR046340**	**OR051734**	**This study**
***A. orienticrocea***	**EFHAAU2919**	**China**	**OR042691**	**OR046336**	**OR051730**	**This study**
***A. orienticrocea***	**EFHAAU4837**	**China**	**OR042692**	**OR046337**	**OR051731**	**This study**
*A. orienticrocea*	HKAS80029	China	MH486700	–	–	[2]
*A. orienticrocea*	HKAS90455	China	MH486701	MH508942	MH486133	[2]
*A. orientifulva*	HKAS32522	China	AY436496	–	–	[51]
*A. orientifulva*	HKAS87937	China	MH486704	MH509154	MH486136	[2]
‘*A. orientifulva*’	KA12-0642	South Korea	KF021679	–	–	[32]
‘*A. orientifulva*’	STDS-2-10	Japan	LC098763	–	–	Direct sub.
*A. ovalispora*	HKAS101406	China	MH486720	MH508955	MH486148	[2]
*A. pachycolea*	HKAS101422	USA	MH486724	–	MH486152	[2]
*A. pallidocarnea*	HKAS97678	China	MH486728	–	MH486156	[2]
***A. pallidozonata***	**EFHAAU114**	**China**	**OR042693**	**–**	**OR051722**	**This study**
***A. pallidozonata***	**EFHAAU1594**	**China**	**OR042694**	**–**	**OR051732**	**This study**
***A. pallidozonata***	**EFHAAU373**	**China**	**OR042695**	**OR046338**	**OR051733**	**This study**
***A. pallidozonata***	**EFHAAU542**	**China**	**OR042696**	**OR046339**	**–**	**This study**
*A. pallidozonata*	HKAS57718	China	MH486740	MH508973	–	[2]
*A. pallidozonata*	HKAS100608	China	MH486739	–	MH486164	[2]
*A. pekeoides*	JAC13244	New Zealand	MT862269	MT977108	MT993777	Direct sub.
*A. populiphila*	RET 068-7	USA	KP221315	–	–	Direct sub.
*A. prudens*	MP220407	Spain	OP279613	–	–	[34]
*A. pseudovaginata*	HKAS70138	China	MH486791	–	MH486205	[2]
*A. retenta*	HKAS70020	China	MH486802	MH509028	MH486215	[2]
*A. shennongjiana*	HKAS75553	China	MH486862	MH509085	MH486270	[2]
*A. shennongjiana*	HKAS75554	China	MH486863	–	–	[2]
*A. simulans*	JM0303	Belgium	–	–	MF440425	[4]
***A. sinofulva***	**EFHAAU207**	**China**	**OR042700**	**OR046342**	**–**	**This study**
***A. sinofulva***	**EFHAAU313**	**China**	**OR042701**	**OR046343**	**–**	**This study**
***A. sinofulva***	**EFHAAU118**	**China**	**OR042699**	**OR046341**	**OR051736**	**This study**
***A. sinofulva***	**HKAS75058**	**China**	**OR042702**	**OR046344**	**OR051737**	**This study**
***A. sinofulva***	**HKAS92355**	**China**	**OR042703**	**OR046345**	**OR051738**	**This study**
*A.* aff. *sinicoflava*	TRTC156849	Canada	ON392637	ON492153	–	[33]
*A.* aff. *sinicoflava*	TRTC156851	Canada	ON392638	ON492154	–	[33]
*A. sororcula*	ANDES_F2088_NVE587	Colombia	KT008030	KT008013	–	[23]
*A. strobilaceovolvata*	JEIC0609	Ivory Coast	ON843372	ON894364	ON855006	[33]
*A. submembranacea*	MB -001174	Germany	MH486916	MH509135	–	[2]
*A. suborientifulva*	BZ2013-55	Thailand	MF461564	–	–	[4]
***A. suborientifulva***	**EFHAAU3559**	**China**	**OR042687**	**–**	**–**	**This study**
*A. suborientifulva*	OR1276	Thailand	MF461567	–	MF440426	[4]
***A*** **. cf. *suborientifulva***	**EFHAAU4437**	**China**	**OR042685**	**OR046332**	**OR051726**	**This study**
***A*** **. cf. *suborientifulva***	**EFHAAU5291**	**China**	**OR042688**	**OR046333**	**OR051727**	**This study**
*A. subovalispora*	BZ2014-06	Thailand	MF461565	–	MF440409	[4]
*A. subovalispora*	BZ2015-70	Thailand	MF461562	–	MF440421	[4]
***A. subovalispora***	**HKAS128053**	**China**	**OR042727**	**OR046363**	**OR051754**	**This study**
***A. subovalispora***	**EFHAAU2621**	**China**	**OR042728**	**–**	**–**	**This study**
***A. subovalispora***	**EFHAAU3558**	**China**	**OR042729**	**–**	**–**	**This study**
***A. subovalispora***	**EFHAAU4075**	**China**	**OR042730**	**–**	**–**	**This study**
***A. subovalispora***	**EFHAAU4480**	**China**	**OR042731**	**OR046364**	**OR051755**	**This study**
***A. subovalispora***	**HKAS128050**	**China**	**OR042732**	**OR046365**	**OR051756**	**This study**
*A. subtropicana*	TM 15-995	India	MG923799	–	–	Direct sub.
*A. sulcatissima*	TRTC176558	Brazil	ON392674	ON540324	ON492094	[33]
*A.* cf. *sulcatissima*	TRTC176754	Brazil	ON470140	ON540342	ON492103	[33]
*A. tenuifulva*	HKAS87120	China	MH486929	MH509146	MH486322	[2]
*A. tenuifulva*	HKAS58877	China	MH486928	MH509145	–	[2]
***A. tomentosivolva***	**HKAS108152**	**China**	**OR042733**	**OR046366**	**OR051758**	**This study**
*A. umbrinolutea*	HKAS89201	China	MH486933	MH509150	MH486326	[2]
*A. umbrinolutea*	MB-000658	Germany	MH486937	–	MH486330	[2]
*A. vaginata* var. *vaginata*	HAvdAasn_Holand	The Netherlands	AF024482	–	–	[49]
*A.* cf. *velosa*	TRTC157486	Canada	ON392642	ON492127	–	[33]
*A. verrucosivolva*	HKAS28253	China	AF024483	–	–	[49]
*A. verrucosivolva*	HKAS75608	China	MH486939	MH509156	MH486332	[2]
*A. vladimirii*	BRNM825829	Czech Republic	MW208921	MW208626	–	[31]
***A. zonata***	**EFHAAU607**	**China**	**OR042734**	**OR046367**		**This study**
***A. zonata***	**EFHAAU709**	**China**	**OR042735**	**OR046368**		**This study**
***A. zonata***	**EFHAAU755**	**China**	**OR042736**	**OR046369**	**OR051757**	**This study**
*A. zonata*	HKAS97240	China	MH486959	MH509179	MH486352	[2]
*A. zonata*	HKAS97244	China	MH486960	MH509180	MH486353	[2]
***A.*** **cf. *zonata***	**EFHAAU2254**	**China**	**OR042686**	**–**		**This study**
*Amanita* sp.	RET 732-8	USA	MT013999	–	–	Direct sub.
*Amanita* sp.	RET 374-3	USA	MN614413	–	–	Direct sub.
*Amanita* sp.	TRTC156902	Canada	ON392647	ON492128	–	[33]
*Amanita* sp.	TRTC176759	Brazil	ON392666	ON540336	ON492124	[33]
*Amanita* sp.	JEIC0513	Guinea	ON843345	ON894338	ON854987	[33]
*Amanita* sp.	JEIC0674	Benin	ON843350	ON894344	ON854993	[33]
*Amanita* sp.	JEIC0737	Benin	ON843357	ON894351	ON854997	[33]
*Amanita* sp.	JEIC0592	Togo	ON843359	ON894353	–	[33]
*Amanita* sp.	JEIC0510	Guinea	ON843360	ON894355	ON854998	[33]
*Amanita* sp.	JEIC0583	Benin	ON843362	ON894357	–	[33]
*Amanita* sp.	JEIC0625	Benin	ON843366	ON894358	ON855001	[33]
*Amanita* sp.	JEIC0599	Togo	ON843367	ON894359	ON855002	[33]
*Amanita* sp.	JEIC0691	Benin	–	ON931616	ON855003	[33]
*Amanita* sp.	INPA No. 265223	Brazil	ON392669	ON492134	ON492118	[33]
*Amanita* sp.	JEIC0723	Benin	ON843369	ON894361	ON855005	[33]
*Amanita* sp.	JEIC0602	Ivory Coast	ON843370	ON894362	–	[33]
*Amanita* sp.	JEIC0598	Togo	ON843371	ON894363	–	[33]
*Amanita* sp	TRTC157487	Canada	ON392648	ON520571	–	[33]
*Amanita* sp.	INPA No. 265290	Brazil	ON392671	ON492136	ON492120	[33]
*Amanita* sp.	TRTC176599	Brazil	ON392673	ON540335	ON492121	[33]
*Amanita* sp.	JEIC0652	Benin	ON843339	–	ON854981	[33]
*Amanita* sp.	JEIC0724	Benin	ON843343	ON894336	ON854985	[33]
**Outgroup**						
*A. caesarea*	HKAS96166	Italy	MH486418	MH508705	MH485898	[2]
*A. muscaria*	MB-001171	Germnay	MH486652	MH508909	MH486101	[2]
*A. parvipantherina*	HKAS54723	China	KR824780	KR824807	KR824802	[53]
*A. yuaniana*	HKAS58807	China	MH486954	MH509174	MH486347	[2]

Quotation marks are added to indicate the uncertain taxonomic positions, – represents missing corresponding sequences.

## 3. Results

### 3.1. Phylogenetic Analyses 

Overall, 163 sequences—including 56 for nrLSU, 42 for *tef1-α*, 37 for *rpb2*, and 28 for ITS—were newly generated in this study, and they were aligned with the sequences downloaded from GenBank. The sequences retrieved from GenBank and obtained in this study are listed in Table 1 and Table S1. The concatenated dataset (nrLSU, *tef1-α*, and *rpb2*) included 429 sequences from 201 samples representing 112 taxa (Table 1). The raw concatenated dataset comprised 2146 positions, and the final matrix retained 1846 positions, with 646 parsimony-informative sites, after excluding introns and poorly aligned regions. In the ITS dataset, 128 sequences from 64 taxa were included (Table S1). The dataset comprised 903 positions, with 384 parsimony-informative sites, and 570 positions of the ambiguously aligned regions were excluded. Six and two subsets were selected for the combined and ITS datasets, respectively. The best partition schemes and corresponding best-fits models are summarized in Table 2. 

**Figure 1:**
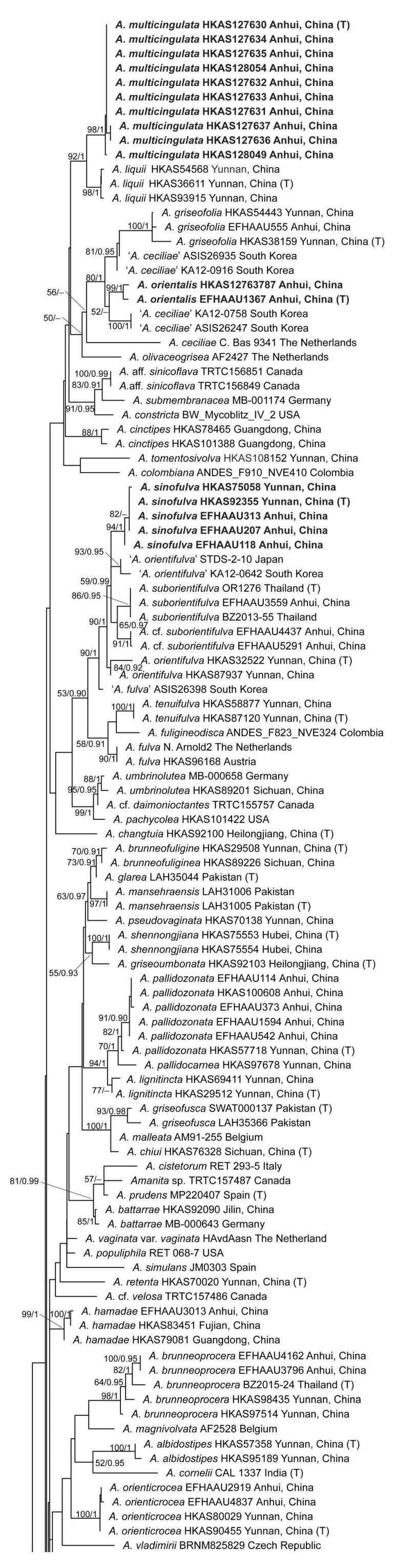

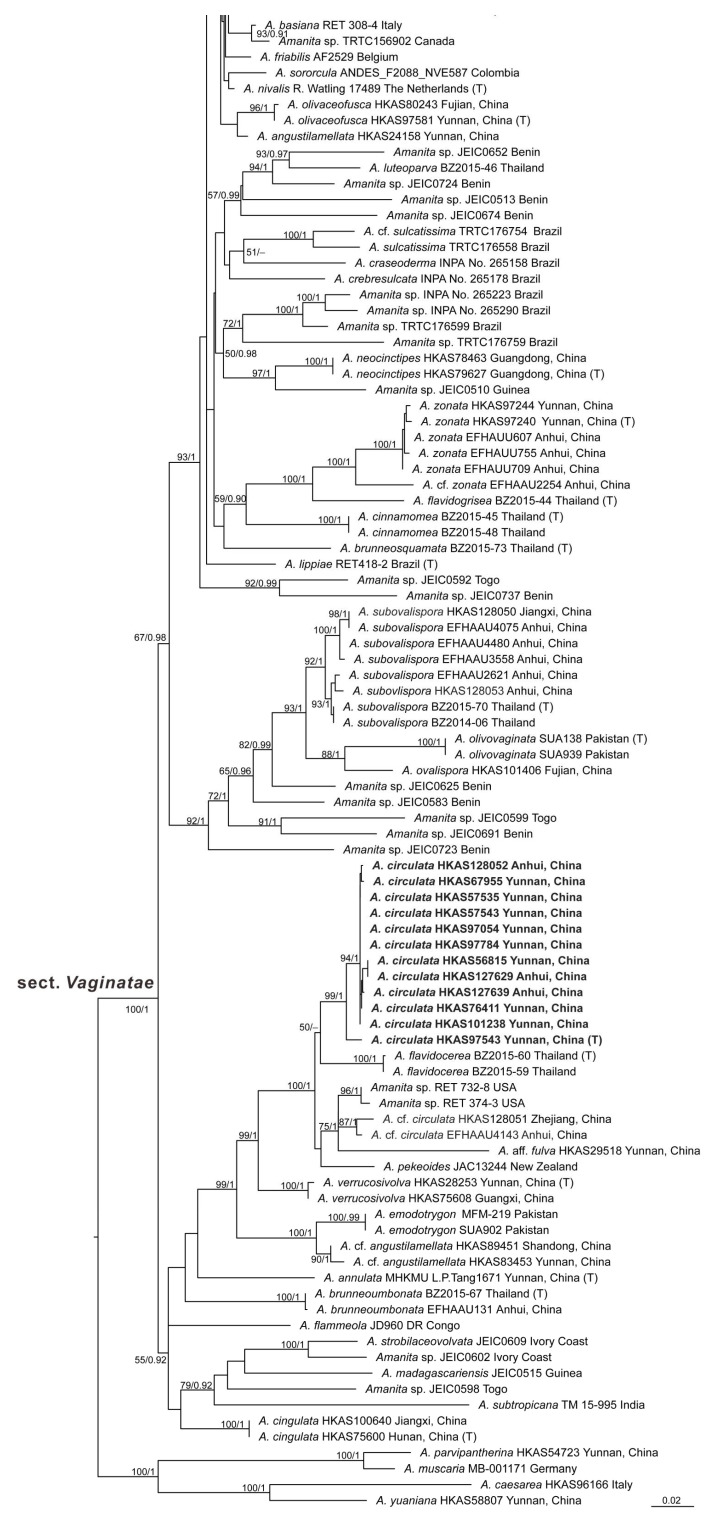
Phylogenetic tree of *Amanita* sect. *Vaginatae* inferred from maximum likelihood analyses based on the combined dataset (nrLSU, *tef1-α* and *rpb2*). Bootstrap values over 50% and Bayesian posterior probabilities over 0.90 are shown along the branches. Sequences from type collections are indicated with (T), and new species are in boldface.

The phylogenetic trees inferred from the ML and BI analyses were similar in topology. Therefore, only the trees obtained from the ML analyses were presented (Figure 1 and Figures S1–S4). In the phylogenetic tree based on the combined matrix, the collections from eastern China were clustered into 19 lineages, including 12 known species and seven undescribed taxa (Figure 1). Four of them were described as new species, namely *A. circulata*, *A. multicingulata*, *A. orientalis*, and *A. sinofulva* (Figure 1 and Figures S1–S4). *Amanita circulata* formed a monophyletic clade with *A. flavidocerea* Thongbai et al. from Thailand, *A. pekeoides* G.S. Ridl. from New Zealand, *A. verrucosivolva* Zhu L. Yang from China, and three undescribed taxa (*A*. aff. *fulva* HKAS29518 China, *Amanita* sp. RET 732-8, and 374-3 USA, *A.* cf. *circulata* China) (Figure 1 and Figure S4). *Amanita multicingulata* was sister to *A. liquii* Zhu L. Yang et al. from China. *Amanita orientalis* formed a monophyletic group with *A. griseofolia* Zhu L. Yang and another two species from South Korea, erroneously identified as *A. ceciliae* (Berk. & Broome) Bas. In the phylogenetic tree inferred from the ITS dataset, the taxon was more closely related to the sample (JL2) from China, which was labeled as *A. griseofolia*, and two collections (SUA441 and SUA510) from Pakistan, with moderate support (Figure S4). *Amanita sinofulva* was clustered in the clade formed by *A. orientifulva* Zhu L. Yang et al., *A. suborientifulva* Raspé et al., and another five collections from China, Japan, and South Korea, which were labeled as *A.* cf. *suborientifulva* (EFHAAU4437 and EFHAAU5291), or erroneously identified as *A. orientifulva* (STDS-2-10 and KA12-0642) and *A. fulva* Fr. (ASIS26398), respectively (Figure 1 and Figure S4). 

The remaining lineages represented three putatively new taxa. As only one or two collections were included in every species, they will be described in the future with adequate samples.

### 3.2. Taxonomy

**Figure 2:**
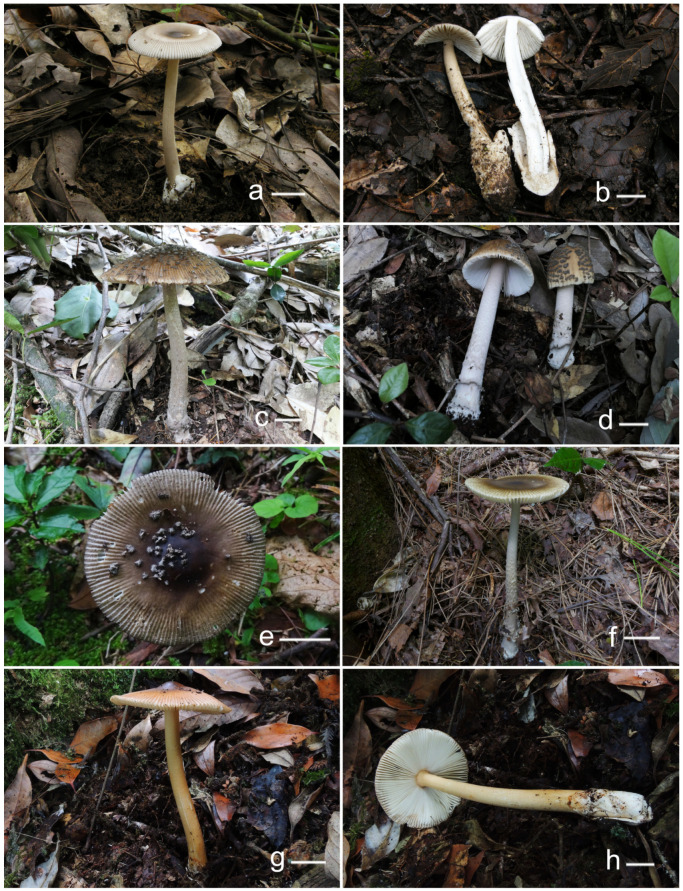
Fresh basidiomata of novel species in *Amanita* sect. *Vaginatae* from eastern China. (**a**,**b**) *A. circulata* ((**a**) HKAS 97784, (**b**) Holotype, HKAS 97543); (**c**,**d**) *A. multicingulata* (Holotype, HKAS 127630); (**e**,**f**) *A. orientalis* ((**e**) HKAS 127638, (**f**) EFHAAU 1367); (**g**,**h**) *A. sinofulva* (Holotype, HKAS92355). Bar = 2 cm.

**Figure 3:**
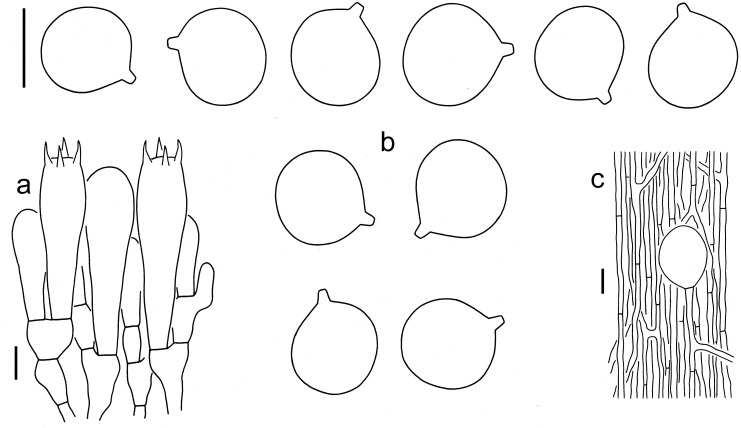
Microscopic features of *A. circulata* (Holotype, HKAS 97543). (**a**). Hymenium and subhymenium; (**b**). Basidiospores; (**c**). Interior of volval remnants on the stipe base. Bars: (**a**,**b**) = 10 µm, (**c**) = 20 µm.

***Amanita circulata*** Y.Y. Cui, Q. Cai and Zhu L. Yang, sp. nov., Figure 1, Figure 2 and Figure 3.

Fungal Names: FN 571585.

Etymology: circulata from circular, referring to its circular zone on the pileus.

Diagnosis: Similar to *A. pallidozonata*, but differs in its more filamentous hyphae in the volval remnants on the stipe base.

Type: CHINA. YUNNAN PROVINCE: Puer, Lancang Lahu Autonomous County, in a broad-leaved forest with trees of Fagaceae, altitude 1780 m, 20 August 2016, LC-LJW 39 (Holotype, HKAS 97543, GenBank Acc. Nos.: nrLSU = OR042725, ITS = OR042765, *rpb2* = OR051752, *tef1-α* = OR046361).

Description: Basidioma small to medium-sized. Pileus 3–7.5 cm diam., convex, plano-convex to applanate, umbonate; surface gray-brown (4E2–4) to dark brown (3F6–8) at center and margin, forming a distinctly pale colored [brown (3D2–4) to brownish (2C2–4)] ring-like zone at proximal end of marginal striations; volval remnants on pileus absent; margin striate (0.2–0.5 R), non-appendiculate; trama white (1A1), unchanging. Lamellae free, crowded, white (1A1); lamellar edges white (1A1); lamellulae truncate, plentiful. Stipe 9–18 cm long × 0.5–1.5 cm diam., slender, subcylindric, slightly tapering upwards, with apex slightly expanded, white (1A1), gray (1B1), brownish (2B2–4) to gray-brown (2C2–4); context white (1A1), hollow in center; basal bulb absent; volva saccate, membranous, both surfaces white (1A1). Annulus absent. Odor indistinct.

Lamellar trama bilateral. Mediostratum 20–40 μm wide, composed of abundant, ellipsoid inflated cells (25–60 × 10–30 μm); filamentous hyphae abundant, 2–8 μm wide; vascular hyphae scarce. Lateral stratum composed of abundant, ellipsoid to fusiform inflated cells (20–40 × 10–25 μm), diverging at an angle of ca. 30° to 60° to mediostratum; filamentous hyphae abundant and 2–7 μm wide. Subhymenium 30–40 μm thick, with 2–3 layers of ellipsoid to fusiform or irregularly arranged cells, 5–10 × 5–10 μm. Basidia 45–60 × 15–20 μm, clavate, 4-spored; sterigmata 5–8 μm long; basal septa lacking clamps. Basidiospores [60/3/3] (10.5–) 11–13 (–13.5) × (9.5–) 10–12.5 (–13) μm, Q = 1–1.15 (–1.21), Qm = 1.08 ± 0.05, globose to subglobose, occasionally broadly ellipsoid, inamyloid, colorless, thin-walled, smooth; apiculus small. Lamellar edge appearing as a sterile strip, composed of subglobose to ellipsoid or sphaeropedunculate inflated cells (15–50 × 10–45 μm), single and terminal or in chains of 2–3, thin-walled, colorless; filamentous hyphae abundant, 2–6 μm wide, irregularly arranged or ± running parallel to lamellar edge. Pileipellis 50–90 μm thick; upper layer (15–40 μm thick) gelatinized, composed of radially arranged to interwoven, thin-walled, colorless, filamentous hyphae 2–5 μm wide; lower layer (35–50 μm thick) composed of radially arranged, filamentous hyphae 3–6 μm wide, colorless to brownish; vascular hyphae scarce. Interior of volval remnants on stipe base composed of longitudinally arranged elements: filamentous hyphae dominant and very abundant, 3–10 μm wide, colorless, thin-walled, branching, anastomosing; inflated cells rare, globose, subglobose, ellipsoid to fusiform, 50–80 × 40–50 μm, colorless, thin-walled, mostly terminal or sometimes in chains of 2–3. Outer and inner surface of volval remnants on stipe base similar to structure of interior part, but with inner surface gelatinized. Stipe trama composed of longitudinally arranged, clavate terminal cells, 80–250 × 15–40 μm; filamentous hyphae scattered to abundant, 2–10 μm wide; vascular hyphae scarce. Clamps absent in all parts of basidioma.

Habitat: Solitary to scattered on soil in subtropical mixed forests with Fagaceae and Pinaceae.

Distribution: known from eastern and southwestern China.

Additional specimens examined: CHINA. ANHUI PROVINCE: Huangshan, in a broad-leaved forest with trees of Fagaceae, altitude 620 m, 13 July 2018, Hong-Yu Chen 32 (HKAS 127629); same location, in a broad-leaved forest with trees of Fagaceae, altitude 610 m, 12 July 2018, Ting Guo 979 (HKAS 127639). YUNNAN PROVINCE: Baoshan, Tengchong, in a mixed forest with trees of *Pinus*, *Quercus* and *Keteleeria*, altitude 1900 m, 20 July 2009, Li-Ping Tang 858 (HKAS 56815); same county, in a forest with trees of *Pinus armandii* Franch. and *Keteleeria fortune* (A. Murray bis) Carrière, altitude 2010 m, 14 August 2010, Qing Cai 391 (HKAS 67955); same city, Changning County, forest type unknown, altitude 2000 m, 25 July 2009, Gang Wu 4 (HKAS 57535); Kunming, Panlong District, in a mixed forest with trees of Fagaceae and Pinaceae, altitude 1990 m, 21 August 2016, Xiao-Xia Ding 111 (HKAS 97054); same city, Wuhua District, in a mixed forest with trees of Fagaceae and Pinaceae, altitude 1990 m, 6 September 2012, Yan-Jia Hao 753 (HKAS 76411); Lincang, Fengqing County, in a mixed forest with trees of Fagaceae and Pinaceae, altitude 1800 m, 26 July 2009, Gang Wu 12 (HKAS 57543); Puer, Lancang Lahu Autonomous County, in a mixed forest with trees of Fagaceae and Pinaceae, altitude 1780 m, 29 September 2016, LC-LJW 280 (HKAS 97784); same county, in a forest dominated with trees of Fagaceae, altitude 1350 m, 31 August 2017, Zhu L. Yang 6049 (HKAS 101238).

Notes: *Amanita circulata* is somewhat related to *A. flavidocerea* (Figure 1). However, the latter can be easily distinguished from the former species by its non-umbonate pileus, which is yellow and lacks a ring-like zone at the proximal end of marginal striations [4]. *Amanita pallidozonata* and *A. zonata* Y.Y. Cui et al. might be confused with *A. circulata* due to the pronounced ring-like zones at the proximal end of the marginal striations. However, *A. pallidozonata* differs from *A. circulata* by its more inflated cells in the inner part of volval remnants on the stipe base [2]. *Amanita zonata* has relatively smaller basidiospores (9–10.5 × 8.5–10 μm) [2]. 

**Figure 4:**
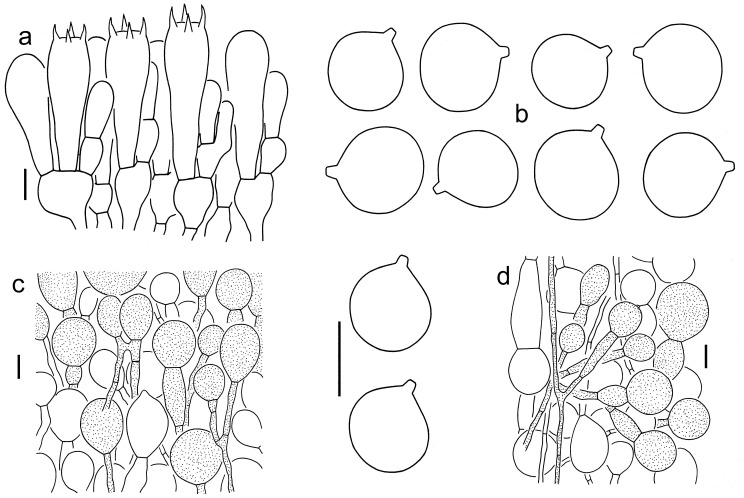
Microscopic features of *A. multicingulata* (Holotype, HKAS 127630). (**a**). Hymenium and subhymenium; (**b**). Basidiospores; (**c**). Volval remnants on the pileus; (**d**) Volval remnants on the stipe base (right side indicates outer part). Bars: (**a**,**b**) = 10 µm, (**c**,**d**) = 20 µm.

***Amanita multicingulata*** Y.Y. Cui, Q. Cai and Zhu L. Yang, sp. nov., Figure 1, Figure 2 and Figure 4.

Fungal Names: FN 571586.

Etymology: multicingulata named after its tomentose volval remnants often arranged in incomplete rings on the stipe base.

Diagnosis: Close to *A. liquii*, but differs in its longer striations on pileal margin, white to dirty white lamellae without obvious color change when dried, smaller basidiospores and distributions in subtropical forests dominated with Fagaceae, sometimes mixed with *Pinus*.

Type: CHINA. ANHUI PROVINCE: Huangshan, in a forest dominated with Fagaceae, altitude 1390 m, 13 July 2018, Ting Guo 1017 (Holotype, HKAS 127630, GenBank Acc. Nos.: nrLSU = OR042713, ITS = OR042750, *rpb2* = OR051745, *tef1-α* = OR046352).

Description: Basidioma small, medium-sized to large. Pileus 3–11 cm diam., plano-convex to applanate, surface gray-brown (2C2–4), brown (3E3–5) to yellow-brown (4C4–6), often darker at center; volval remnants on pileus verrucose to felted, dark gray (1E1–3) to gray (1C1–3), often dirty white (1B1) at apical part; margin striate (0.3–0.6 R), non-appendiculate; trama white (1A1) to dirty white (1B1), unchanging. Lamellae free, crowded, white (1A1) to dirty white (1B1), sometimes with brownish (3B2–3) tinge; lamellar edges gray (1B1), gray-brown (1B2) to brown (3B2–4); lamellulae truncate, plentiful. Stipe 10–17 cm long × 0.5–1.5 cm diam., slender, subcylindric, slightly tapering upwards, with apex slightly expanded, grayish (1B1), brownish (1B2) to gray-brown (2D2–4), covered with concolor squamules; context white (1A1) to dirty white (1B1), hollow in center; basal bulb absent; volval remnants on stipe base tomentose, arranged in incomplete rings, gray (1C1–3) to gray-brown (1B2). Annulus absent. Odor indistinct.

Lamellar trama bilateral. Mediostratum 20–30 μm wide, composed of abundant, clavate inflated cells (50–80 × 10–20 μm); filamentous hyphae abundant, 2–8 μm wide; vascular hyphae scarce. Lateral stratum composed of abundant, ellipsoid to fusiform inflated cells (20–40 × 10–20 μm), diverging at an angle of ca. 30° to 60° to mediostratum; filamentous hyphae abundant and 2–8 μm wide. Subhymenium 30–50 μm thick, with 2–3 layers of ellipsoid to fusiform or irregularly arranged cells, 10–30 × 8–20 μm. Basidia 40–60 × 13–18 μm, clavate, 4-spored; sterigmata 5–8 μm long; basal septa lacking clamps. Basidiospores [40/2/2] (9.5–) 10–12 (–12.5) × (9–) 9.5–11 (–11.5) μm, Q = 1–1.13 (–1.15), Qm = 1.06 ± 0.03, globose to subglobose, inamyloid, colorless, thin-walled, smooth; apiculus small. Lamellar edge appearing as a sterile strip, composed of subglobose, ellipsoid to clavate inflated cells (10–45 × 10–30 μm), single and terminal or in chains of 2–3, thin-walled, colorless; filamentous hyphae abundant, 2–8 μm wide, irregularly arranged or ± running parallel to lamellar edge. Pileipellis 50–100 μm thick; upper layer (30–50 μm thick) gelatinized, composed of radially arranged to interwoven, thin-walled, colorless filamentous hyphae 2–5 μm wide; lower layer (40–50 μm thick) composed of radially arranged filamentous hyphae 4–7 μm wide, colorless; vascular hyphae scarce. Volval remnants on pileus composed of more or less vertically arranged elements: inflated cells very abundant to dominant, globose, subglobose, ellipsoid to fusiform, 10–60 × 10–50 μm, brown to brownish or colorless, thin-walled, mostly terminal or sometimes in chains of 2–3; filamentous hyphae rare, 3–7 μm wide, brown to brownish or colorless, thin-walled, branching, anastomosing. Volval remnants on stipe base composed of longitudinally arranged elements, becoming horizontally arranged towards upper parts: inflated cells very abundant to nearly dominant, globose, subglobose, ellipsoid, fusiform to clavate, 20–80 × 10–50 μm, brown to brownish or colorless, thin-walled, mostly terminal or sometimes in chains of 2–3; filamentous hyphae rare to fairly abundant, 2–8 μm wide, brown to brownish or colorless, thin-walled, branching, anastomosing. Stipe trama composed of longitudinally arranged, clavate terminal cells, 100–400 × 15–40 μm; filamentous hyphae scattered to abundant, 2–10 μm wide; vascular hyphae scarce. Clamps absent in all parts of basidioma.

Habitat: Solitary to scattered on soil in subtropical broad-leaved forests dominated with Fagaceae, sometimes in mixed forests with fagaceous and *Pinus* plants.

Distribution: Known from eastern China.

Additional specimens examined: CHINA. ANHUI PROVINCE: Huangshan, in a forest dominated with Fagaceae, altitude 1300 m, 13 July 2018, Ting Guo 1018 (HKAS 127631); same location, in a forest dominated with Fagaceae, altitude 670 m, 13 July 2018, Rui-Heng Yang 73 (HKAS 127632); same location, in a forest with Fagaceae and Pinaceae, altitude 940 m, 15 July 2018, Rui-Heng Yang 123 (HKAS 127633); same location, in a forest dominated with Fagaceae, altitude 760 m, 15 July 2018, Rui-Heng Yang 127 (HKAS 127634); same location, in a forest dominated with Fagaceae, altitude 1220 m, 17 July 2018, Rui-Heng Yang 184 (HKAS 127635); same location, in a forest dominated with Fagaceae, altitude 1200 m, 17 July 2018, Rui-Heng Yang 186 (HKAS 127636); same location, in a forest dominated with Fagaceae, altitude 940 m, 15 July 2018, Hong-Yu Chen 77 (HKAS 127637).

Notes: Based on molecular phylogenetic analysis, *A. multicingulata* is closely related to *A. liquii* (Figure 1 and Figure S4), but the latter species has shorter striations on pileal margin (0.1–0.3 R), larger basidiospores (11.5–15 × 11–14.5 μm) and occurs in subalpine forests dominated by trees of *Picea* and *Abies* [2,35,54]. In addition, *A. liquii* has white to grayish lamellae that turn dark gray to dark brown when dried [2,35,54]. *Amanita cinctipes* Corner and Bas, *A. griseofolia* and *A. neocinctipes* Zhu L. Yang et al. of *A.* sect. *Vaginatae* with a nonsaccate volva were also reported from China. Smaller basidiospores (9–10.5 × 8–9.5 μm) and shorter striations on the pileal margin (0.3–0.4 R) distinguish *A. cinctipes* from *A. multicingulata* [2,29]. *Amanita griseofolia* differs from *A. multicingulata* by its more grayish pileus and slightly larger basidiospores (10–13.5 × 9.5–13 μm) [1,2,55]. White lamellae and subglobose to broadly ellipsoid basidiospores (8.0–10.5 × 7.0–9.0 μm, Q = 1.09–1.29, Qm = 1.19 ± 0.07) in *A. neocinctipes* set it apart from *A. multicingulata* [2].

**Figure 5:**
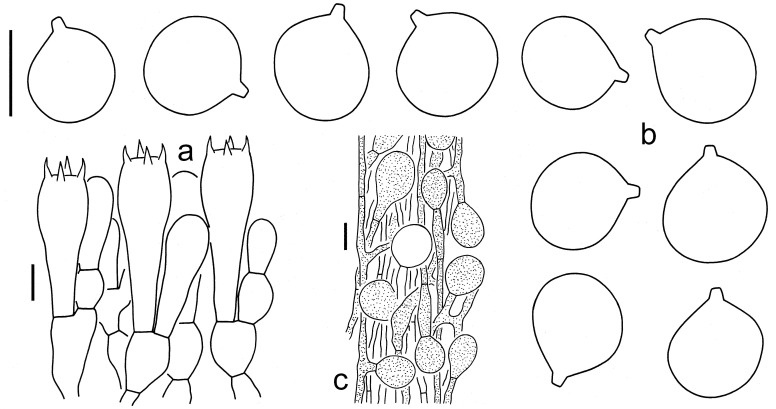
Microscopic features of *A. orientalis* (Holotype, EFHAAU 1367). (**a**). Hymenium and subhymenium; (**b**). Basidiospores; (**c**). Interior of volval remnants on the stipe base (right side indicates outer part). Bars: (**a**,**b**) = 10 µm, (**c**) = 20 µm.

***Amanita orientalis*** Q. Cai, Y.Y. Cui and Zhu L. Yang, sp. nov., Figure 1, Figure 2 and Figure 5.

Fungal Names: FN 571587

Etymology: orientalis means eastern, namely after its type locality from East Asia.

Diagnosis: Close to *A. griseofolia* but differs in its more brownish pileus and mostly subglobose to broadly ellipsoid basidiospores.

Type: CHINA. ANHUI PROVINCE: Huangshan, in a mixed forest with Fagaceae and Pinaceae, altitude 860 m, 15 September 2018, Guo-Qi Chu 153 (Holotype, EFHAAU 1367, GenBank Acc. Nos.: nrLSU = OR042698, ITS = OR042759, *rpb2* = OR051735).

Description: Basidioma small to medium-sized. Pileus 5–7 cm diam., plano-convex to applanate, surface gray-brown (3E3–4) to brown (3D2–4), often darker at center; volval remnants on pileus verrucose to felted, gray (3E1–3), often dirty white (1B1) at apical part; margin striate (0.3–0.5 R), non-appendiculate; trama white (1A1), unchanging. Lamellae free, crowded, white (1A1); lamellar edges white (1A1) to slightly grayish (1B1); lamellulae truncate, plentiful. Stipe 8.5–14.5 cm long × 0.5–1 cm diam., slender, subcylindric, slightly tapering upwards, with apex slightly expanded, dirty white, gray (1B1) to brownish (1B2), covered with gray (1B1) to gray-brown (3C1–3) squamules; context white (1A1), hollow in center; basal bulb absent; volval remnants on stipe tomentose, arranged in incomplete rings, gray (1B1) to gray-brown (3D2–4). Annulus absent. Odor indistinct.

Lamellar trama bilateral. Mediostratum 15–30 μm wide, composed of abundant, clavate inflated cells (30–60 × 10–20 μm); filamentous hyphae abundant, 2–7 μm wide; vascular hyphae scarce. Lateral stratum composed of abundant, ellipsoid to fusiform inflated cells (5–15 × 5–15 μm), diverging at an angle of ca. 30° to 45° to mediostratum; filamentous hyphae abundant and 2–8 μm wide. Subhymenium 20–45 μm thick, with 2–3 layers of ellipsoid to fusiform or irregularly arranged cells, 10–25 × 8–20 μm. Basidia 40–65 × 15–18 μm, clavate, 4-spored; sterigmata 4–6 μm long; basal septa lacking clamps. Basidiospores [40/2/2] (10–) 10.5–13 × 9–12 (–13) μm, Q = 1–1.26 (–1.31), Qm = 1.13 ± 0.07, subglobose to broadly ellipsoid, sometimes globose, inamyloid, colorless, thin-walled, smooth; apiculus small. Lamellar edge appearing as a sterile strip, composed of subglobose, ellipsoid to clavate inflated cells (10–50 × 10–30 μm), single and terminal or in chains of 2–3, thin-walled, colorless; filamentous hyphae abundant, 3–7 μm wide, irregularly arranged or ± running parallel to lamellar edge. Pileipellis 50–100 μm thick; upper layer (30–50 μm thick) gelatinized, composed of radially arranged to interwoven, thin-walled, colorless to brownish filamentous hyphae 2–6 μm wide; lower layer (30–40 μm thick) composed of radially arranged filamentous hyphae 3–8 μm wide, brownish to brown; vascular hyphae scarce. Volval remnants on stipe base composed of longitudinally arranged elements: inflated cells abundant, globose, subglobose, ellipsoid, fusiform to clavate, 20–40 × 10–40 μm, yellow-brown to gray-brown, thin-walled, mostly terminal or sometimes in chains of 2–3; filamentous hyphae abundant, 2–7 μm wide, yellow-brown to gray-brown, thin-walled, branching, anastomosing. Stipe trama composed of longitudinally arranged, clavate terminal cells, 80–300 × 15–40 μm; filamentous hyphae scattered to abundant, 2–10 μm wide; vascular hyphae scarce. Clamps absent in all parts of basidioma.

Habitat: Solitary to scattered on soil in subtropical forests with Fagaceae and Pinaceae.

Distribution: Known from eastern China.

Additional specimens examined: CHINA. ANHUI PROVINCE: Huangshan, in a mixed forest with Fagaceae and Pinaceae, altitude 760 m, 14 July 2018, Rui-Heng Yang 117 (HKAS 127638).

Notes: By having a more grayish pileus and globose to subglobose basidiospores (10–13.5 × 9.5–13 μm, Q = 1.0–1.1, Qm = 1.04 ± 0.03), *A. griseofolia* can be distinguished from *A. orientalis* [2,35,55]. Due to the similarity of their volval remnants on the base of the stipe, the Chinese records of *A. multicingulata*, *A. cinctipes* Corner and Bas, *A. neocinctipes*, and *A. liquii* can be confused with *A. orientalis*. Nevertheless, *A. multicingulata* differs from *A. orientalis* by having basidiospores that are rounder (globose to subglobose, 10–12 × 9.5–11 μm, Q = 1–1.13, Qm = 1.06 ± 0.03). *Amanita cinctipes* differs from *A. orientalis* by its more grayish pileus and smaller and rounder basidiospores (9–10.5 × 8–9.5 μm, Q = 1.0–1.16, Qm = 1.08 ± 0.04) [2,29,35]. The more grayish pileus and smaller basidiospores (8–10.5 × 7–9 μm) of *A. neocinctipes* distinguish it from *A. orientalis* [2]. *Amanita liquii* can be distinguished from *A. orientalis* by its larger basidioma, dark brown to black pileus with shorter striations on its margin (0.1–0.3 R), white to grayish lamellae that turn dark gray when dried, larger and rounded basidiospores (11.5–15 × 11–14.5 μm, Q = 1.0–1.09, Qm = 1.05 ± 0.04), and distribution in alpine to subalpine forests [2,35,54].

Multi-locus phylogenetic research reveals that *A. griseofolia* and the South Korean ‘*A. ceciliae*’ are relatives of *A. orientalis* (Figure 1). The European *A. ceciliae* can be distinguished from *A. orientalis* by its robust basidioma with a brown pileus with a yellow tint and globose to subglobose basidiospores [1,35,56,57,58,59]. Phylogenetically, the aforementioned three taxa seem to be close but clearly different (Figure 1).

**Figure 6:**
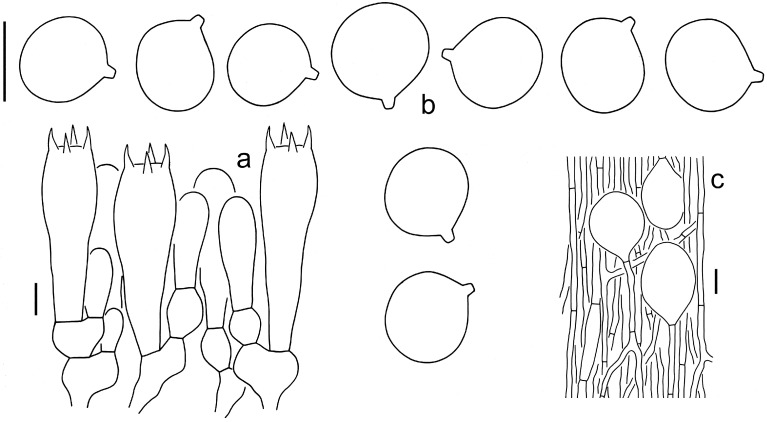
Microscopic features of *A. sinofulva* (Holotype, HKAS 92355). (**a**). Hymenium and subhymenium; (**b**). Basidiospores; (**c**). Interior of volval remnants on the stipe base. Bars: (**a**,**b**) = 10 µm, (**c**) = 20 µm.

***Amanita sinofulva*** Q. Cai, Y.Y. Cui and Zhu L. Yang, sp. nov., Figure 1, Figure 2 and Figure 6.

Fungal Names: FN 571588.

Etymology: sinofulva refers to the fact that this species is found in China and resembles *A. fulva*.

Diagnosis: Close to *A. orientifulva* and *A. suborientifulva*, but *A. orientifulva* has slightly narrower basidiospores and grows in subalpine forests. *Amanita suborientifulva* has a non-umbonate pileus and globose to subglobose or broadly ellipsoid basidiospores.

Type: CHINA. YUNNAN PROVINCE: Dali, Nanjian Yizu Autonomous County, in a broad-leaved forest with trees of Fagaceae, altitude 2515 m, 27 June 2015, Kuan Zhao 727 (Holotype, HKAS 92355, GenBank Acc. Nos.: nrLSU = OR042703, ITS = OR042741, *rpb2* = OR051738, *tef1-α* = OR046345).

Description: Basidioma small to medium-sized. Pileus 3–9 cm diam., plano-convex to applanate, umbonate at center, surface brown (4E6–8) to yellow-brown (5D6–8), often darker at center; volval remnants on pileus absent; margin striate (0.2–0.5 R), non-appendiculate; trama white (1A1), unchanging. Lamellae free, crowded, white (1A1); lamellar edges white (1A1), brownish (4B3–5) to brown (5B3–4); lamellulae truncate, plentiful. Stipe 6–18 cm long × 0.5–1.5 cm diam., slender, subcylindric, slightly tapering upwards, with apex slightly expanded, brown (5B3–4) to brownish (4B3–5); context white (1A1), hollow in center; basal bulb absent; volva saccate, membranous, outer surface white (1A1) with yellow-brown (4B2–4) stains, often yellow-brown (4B2–4) at upper margin, inner surface brownish (5B2–4). Annulus absent. Odor indistinct.

Lamellar trama bilateral. Mediostratum 20–40 μm wide, composed of abundant, fusiform, ellipsoid to clavate inflated cells (25–100 × 10–30 μm); filamentous hyphae abundant, 2–10 μm wide; vascular hyphae scarce. Lateral stratum composed of abundant, ellipsoid to fusiform inflated cells (5–15 × 5–15 μm), diverging at an angle of ca. 30° to 60° to mediostratum; filamentous hyphae abundant and 3–8 μm wide. Subhymenium 30–50 μm thick, with 2–3 layers of ellipsoid to fusiform or irregularly arranged cells, 5–20 × 5–20 μm. Basidia 50–70 × 15–20 μm, clavate, 4-spored; sterigmata 6–8 μm long; basal septa lacking clamps. Basidiospores [40/2/2] (10.5–) 11–13.5 (–18) × (9–) 9.5–12 (–13) μm, Q = (1.01–) 1.05–1.23 (–1.34), Qm = 1.14 ± 0.08, subglobose to broadly ellipsoid, occasionally globose or ellipsoid, inamyloid, colorless, thin-walled, smooth; apiculus small. Lamellar edge appearing as a sterile strip, composed of subglobose, ellipsoid to clavate inflated cells (10–30 × 10–25 μm), single and terminal or in chains of 2–3, thin-walled, colorless; filamentous hyphae abundant, 2–8 μm wide, irregularly arranged or ± running parallel to lamellar edge. Pileipellis 50–90 μm thick; upper layer (30–50 μm thick) gelatinized, composed of radially arranged to interwoven, thin-walled, colorless to brownish filamentous hyphae 2–5 μm wide; lower layer (35–50 μm thick) composed of radially arranged filamentous hyphae 3–7 μm wide, colorless to brownish; vascular hyphae scarce. Interior of volval remnants on stipe base composed of longitudinally arranged elements: filamentous hyphae dominant and very abundant, 3–7 μm wide, colorless, thin-walled, branching, anastomosing; inflated cells rare to fairly abundant, subglobose, ellipsoid to fusiform, 40–65 × 15–50 μm, colorless, thin-walled, mostly terminal or sometimes in chains of 2–3. Stipe trama composed of longitudinally arranged, clavate terminal cells, 80–300 × 15–50 μm; filamentous hyphae scattered to abundant, 3–10 μm wide; vascular hyphae scarce. Clamps absent in all parts of basidioma.

Habitat: Solitary to scattered on soil in subtropical forests dominated with Fagaceae, sometimes mixed with *Pinus*.

Distribution: Known from eastern, central, and southwestern China. Based on the phylogenetic tree inferred from the ITS dataset, it also occurs in Tibet autonomous region and Hunan province (Figure S4). 

Additional specimens examined: CHINA. ANHUI PROVINCE: Liuan, Jinzhai County, in a forest dominated with Fagaceae, altitude 1110 m, 21 July 2017, Yan-Jia Hao 1520 (HKAS 100610); same county, in a mixed forest with *Castanea seguinii* Dode and *Pinus taiwanensis* Hayata, altitude 840 m, Yan-Jia Hao 1609 (EFHAAU 207); same county, in a mixed forest with Fagaceae and Pinaceae, altitude 1000 m, Yan-Jia Hao 1715 (EFHAAU 313). YUNNAN PROVINCE: Nujiang Lisu Autonomous Prefecture, Lanping Bai and Pumi Autonomous County, in a subtropical forest dominated with *Quercus*, mixed with *Pinus yunnanensis*, altitude 2150 m, Gang Wu 743 (HKAS 75058).

Notes: *Amanita orientifulva* and *A. suborientifulva* can be confused with *A. sinofulva*. According to our multi-gene phylogenetic analysis (Figure 1), the first two species also share close relationships with *A. sinofulva*. However, *A. orientifulva* has slightly narrower basidiospores (10.0–14.0 × 9.5–13.0 μm, Q = 1.0–1.12, Qm = 1.06 ± 0.04) and is found in subalpine forests dominated by trees of *Abies* and *Picea* [2,54]. The non-umbonate pileus and globose to subglobose or broadly ellipsoid basidiospores of *A. suborientifulva* set it apart from *A. sinofulva* [4]. The European *A. fulva* is also similar to *A. sinofulva*, but differs in the globose to subglobose basidiospores and in the saccate volva, with inflated cells dominant in its outer part [1,2].

## 4. Discussion

### 4.1. Species Delimitation and Recognition within Amanita sect. Vaginatae

Our data revealed that several macro- and microscopic characteristics could be useful for the delimitation of species in *A.* sect. *Vaginatae*. Five of them are most informative, viz. the color of the basidiomata, the striations on the pileal margin, the presence or absence of the annulus, the volval remnants on the stipe base, and the size of the basidiospores. In this study, *A. cingulata* is the only species with a white basidioma, while the other species from eastern China have basidiomata ranging from yellow, to gray, to brown. The striations on the pileal margin of *A. zonata*, *A. pallidozonata*, and *A. circulata* form a ring-like zone at the proximal end, while the remaining taxa in eastern China are without this zone [2]. Most species in the section are ringless, with only seven taxa with an annulus [12,21,50]. The volval remnants on the stipe base of several species are saccate, while some of them are tomentose, arranged in incomplete rings, viz. *A. griseofolia*, *A. multicingulata*, and *A. orientalis*. Species with an annulus, a ring-like zone at the proximal end, or with incomplete rings of volval remnants on the stipe base are clustered in non-monophyletic groups. 

Given that it is difficult to delimitate these species based solely on morphological studies, integrative taxonomy is indispensable in recognizing species of the section. This method, which delimits and describes taxa by integrating information from different types of data and methodologies (e.g., phylogeny, comparative morphology, habitat and preference of hosts, and behavior), is proven to be useful for species recognition in plants, animals, and fungi [2,60,61,62,63,64]. In this study, species with similar morphological characteristics are successfully recognized using this method. 

For example, *A. zonata*, *A. pallidozonata*, and *A. circulata* are morphologically similar due to the pronounced ring-like zones at the proximal end of the marginal striations. However, they occupy different positions in the phylogenetic tree and are distantly related (Figure 1). Following detailed morphological studies, *A. pallidozonata* can be distinguished from *A. circulata* by its more inflated cells in the inner part of the volval remnants on the stipe base [2]. *Amanita zonata* differs from *A. circulata* by its smaller basidiospores (9–10.5 × 8.5–10 μm) [2]. 

*Amanita sinofulva* is phylogenetically close and morphologically similar to *A. orientifulva*. However, they are clustered into two independent lineages (Figure 1 and Figure S4), and differ in their geographic distributions and host plants. The former species is restricted to the subtropical forests dominated by the trees of Fagaceae and *Pinus*, while the latter is found in the subalpine forests under *Picea* spp., *Abies* spp., and *Quercus* spp. [2,54]. Furthermore, the latter can be distinguished from the former by its narrower basidiospores (10–14 × 9.5–13 μm).

Overall, the combination of morphological characteristics, multi-locus phylogeny, and ecological data can make the result of species delimitation more reliable and objective.

### 4.2. Phylogenetic Relationships of Amanita sect. Vaginatae Species in Southeast Asia and Southern Parts of China

In this study, 40 taxa of *A.* sect. *Vaginatae* were delimitated in China, including 33 known taxa [1,2,21], four species new to sciences (*A. circulata*, *A. multicingulata*, *A. orientalis* and *A. sinofulva*), and three species new to China (*A. brunneoumbonata* Thongbai et al., *A. suborientifulva* and *A. subovalispora* Thongbai et al.). Thirty-nine of them are reported from the southern parts of China—namely, southwestern, central, eastern, and southern China. 

According to our phylogenetic analyses, species of the section from the southern parts of China are closely related to those reported from Southeast Asia. For example, of the 13 species reported from Southeast Asia [4,29,65,66], 7 of them also occur in the southern parts of China (Figure 1). Among them, *A. angustilamellata* (Höhn.) Boedijn, *A. brunneoprocera* Thongbai et al., *A. cinctipes*, and *A. pallidocarnea* (Höhn.) Boedijn are typical tropical elements restricted in the tropical areas of China. The other three species, viz. *A. brunneosquamata* Thongbai et al., *A. suborientifulva*, and *A. subovalispora*, extend their distribution from Southeast Asia to subtropical China. In addition, several taxa found in subtropical or subalpine temperate areas in southern parts of China are phylogenetically close to species from Southeast Asia. For example, *A. circulata* and *A. zonata*, reported from the subtropical regions of China, are sister to *A. flavidocerea* and *A. flavidogrisea* Thongbai et al. from Southeast Asia, respectively (Figure 1). *Amanita pallidozonata* from the subtropical areas and *A. orientifulva* from the subalpine forests of the southern parts of China are closely related to *A. pallidocarnea* from Southeast Asia and tropical China (Figure 1). Therefore, the species in the southern parts of China may have historical affinities in common with those of tropical Asia [67]. This was also consistent with the results of Codjia [33], in which part of the taxa in East Asia were indicated to have migrated from Southeast Asia.

Previously, only five species of *A.* sect. *Vaginate* were reported from eastern China [2]. In this study, 16 species were delimitated, with four new species and 12 newly recorded species. For the convenience of recognition, a key to them is provided.

**Key to the Species of *Amanita* sect. *Vaginatae* from Eastern China**

1. Basidioma white; volval remnants on pileus present as patches; annulus present; basidiospores mostly ellipsoid to elongate…………….……………….…….…..………*A. cingulata*
1’. Basidioma yellow, gray to brown; volval remnants on pileus usually absent, sometimes present as verrucae to felts; basidiospores mostly globose to subglobose, sometimes broadly ellipsoid………………………………………….….……………………….…………..2
2. Volval remnants on pileus mostly present as verrucae to felts; volval remnants on stipe base tomentose, arranged in incomplete rings………………………….……….…………….3
2’. Volval remnants on pileus often absent; volval remnants on stipe base saccate………………………………………………………………………………………………....5
3. Basidiospores subglobose to broadly ellipsoid, 10.5–13 × 9–12 μm, Q = 1–1.26 (–1.31), Qm = 1.13 ± 0.07……………………………….………………………………………*A. orientalis*
3’. Basidiospores more rounded, globose to subglobose……………………………………..4
4. Basidioma more grayish; basidiospores slightly larger, 10–13.5 × 9.5–13 μm………………………………………………………………..……….…….……*A. griseofolia*
4’. Basidioma more brownish; basidiospores slightly smaller, 10–12 × 9.5–11 μm………………………………………………………………….……………*A. multicingulata*
5. Pileal surface forming a distinctive ring-like zone at proximal end of marginal striations………………………………….…………………………………..………………………...6
5’.v Pileal surface without a distinctive ring-like zone at proximal end of marginal striations………………………………….……………………………………………..……………...8
6. Pileal margin with relatively shorter striations, 0.15–0.3 R; basidiospores slightly smaller and rounder, globose to subglobose, 9–10.5 × 8.5–10 μm, Q = 1.00–1.11, Qm = 1.05 ± 0.04……………………………………...……………………………………..………….*A. zonata*
6’. Pileal margin with relatively longer striations; basidiospores slightly larger, globose, subglobose to broadly ellipsoid……………………….………………………………………...7
7. Basidiospores slightly smaller, 10–12 × 9–11 μm; volval remnants on stipe base with abundant inflated cells in inner part……………………………………………*A. pallidozonata*
7’. Basidiospores slightly larger, 11–13 × 10–12.5 μm; volval remnants on stipe base mainly with abundant filamentous hyphae………………………….……………………...*A. circulata*
8. Basidioma with distinctive yellow color…………………...…………………………..…….9
8’. Basidioma gray to brown, without yellow color…………………………………………..10
9. Striations on pileal margin relatively longer, 0.2–0.5 R; basidiospores broadly ellipsoid to ellipsoid, 10–12 × 8–9 μm, Q = 1.22–1.37, Qm = 1.3 ± 0.07……………….………*A. hamadae*
9’. Striations on pileal margin relatively shorter, 0.2–0.3 R; basidiospores globose to subglobose, 9–11 × 9–11 μm, Q = 1–1.11, Qm = 1.05 ± 0.04……………………………….*A. crocea*
10. Basidioma orange-brown to yellow-brown…………………….…………….…………..11
10’. Basidioma gray to gray-brown………………………………………………….………...12
11. Pileus without an umbo……………………………...……….……………*A. suborientifulva*
11’. Pileus with a distinctive umbo at center……………...…………………...…...*A. sinofulva*
12. Pileus gray-brown to yellow-brown, with distinct olivaceous tinge; basidiospores broadly ellipsoid to ellipsoid, relatively larger, 10.5–13 × 8.5–10 μm, Q = 1.05–1.45, Qm = 1.25 ± 0.09………………………….………………………………………………*A. olivaceofusca*
12’. Pileus gray to brown, without olivaceous tinge; basidiospores globose to subglobose, or broadly ellipsoid to ellipsoid, relatively smaller…………………………………………13
13. Pileus gray, without umbo at the center; basidiospores broadly ellipsoid to ellipsoid……………………………………………………………………………….………………14
13’. Pileus gray-brown to brown, umbonate at center; basidiospores globose to subglobose………………………………………………………………………………………….……15
14. Pileal margin with shorter striations, 0.36–0.4 R………………………….*A. subovalispora*
14’. Pileal margin with longer striations, 0.4–0.6 R………...………………….......*A. ovalispora*
15. Pileus dark brownish, with darker colored central disk; pileal margin with longer striations, 0.33–0.42 R…………………………………………………………..*A. brunneoumbonata*
15’. Pileus grayish brown; pileal margin with shorter striations, 0.18–0.21 R………………………………………………………………………………..*A. brunneoprocera*

## Figures and Tables

**Table 2:** The best partition schemes and models selected by PartitionFinder.

Subsets in the Best-Fit Partition Scheme	The Base Positions of Each Subset	Best-Fit Model
nrLSU	1–775	TIM+I+G
*tef1-α*_condon1	776–1184\3	TVMef+I+G
*tef1-α*_condon2, rpb2_condon3	777–1184\3, 1187–1846\3	SYM+I+G
tef1-α_condon3	778–1184\3	TIM+I+G
rpb2_condon1	1185–1846\3	GTR+I+G
rpb2_condon2	1186–1846\3	HKY+I+G
ITS1, ITS2	1–92, 250–333	HKY+I+G
5.8S	94–249	TIMef+I+G

## Data Availability

Publicly available datasets were analyzed in this study (https://www.ncbi.nlm.nih.gov/, accessed on 1 May 2023; https://nmdc.cn/fungalnames/, accessed on 25 May 2023).

## Supplementary Materials

The following supporting information can be downloaded at: https://www.mdpi.com/article/10.3390/jof9080862/s1, Figure S1: Phylogenetic tree of *Amanita* sect. *Vaginatae* inferred from maximum likelihood analyses based on the nrLSU sequences. Bootstrap values over 50% are shown along the branches. Sequences from type collections are indicated with (T), and new species are in boldface; Figure S2: Phylogenetic tree of *Amanita* sect. *Vaginatae* inferred from maximum likelihood analyses based on the *tef1-α* sequences. Bootstrap values over 50% are shown along the branches. Sequences from type collections are indicated with (T), and new species are in boldface; Figure S3: Phylogenetic tree of *Amanita* sect. *Vaginatae* inferred from maximum likelihood analyses based on the *rpb2* sequences. Bootstrap values over 50% are shown along the branches. Sequences from type collections are indicated with (T), and new species are in boldface; Figure S4: Phylogenetic tree of *Amanita* sect. *Vaginatae* inferred from maximum likelihood analyses based on the ITS sequences. Bootstrap values over 50% and Bayesian posterior probabilities over 0.90 are shown along the branches. Sequences from type collections are indicated with (T), and new species are in boldface; Table S1: Voucher information and GenBank accession numbers of the samples used in the phylogenetic analyses of ITS sequences. Sequences newly generated in this study are indicated in bold.
