# Supplementary material for: Species Diversity of Amanita Section Vaginatae in Eastern China, with a Description of Four New Species
Source: J Fungi (Basel). 2023 Aug 19;9(8):862. doi: 10.3390/jof9080862 (PMC10456051; doi:10.3390/jof9080862)
Supplement: Supplementary file 1 [file jof-09-00862-s001.zip › Figures S1-S4.pdf]

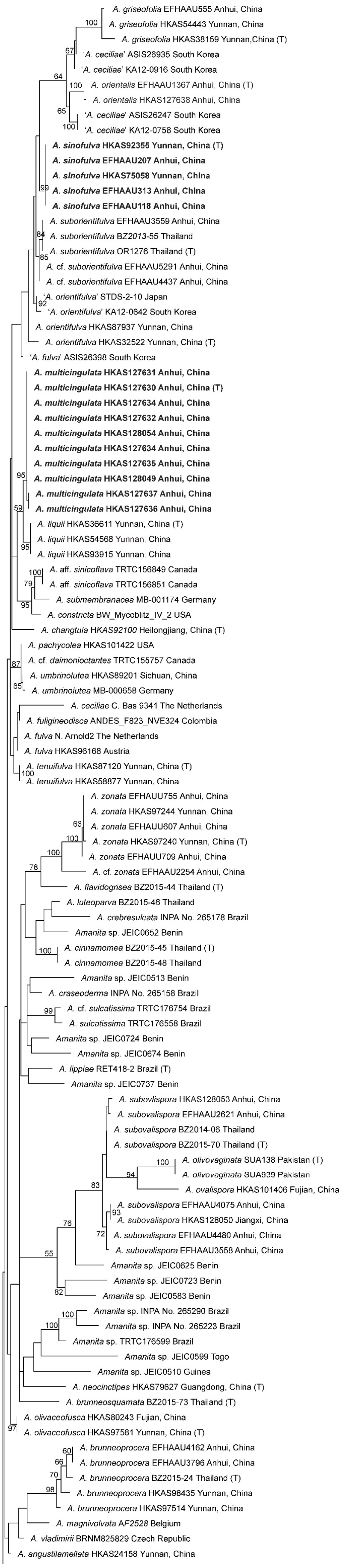
A. griseofolia EFHAAU555 Anhui, China
A. griseofolia HKAS54443 Yunnan, China
A. griseofolia HKAS38159 Yunnan,China (T)
'A. ceciliae' ASIS26935 South Korea
'A. ceciliae' KA12-0916 South Korea
A. orientalis EFHAAU1367 Anhui, China (T)
A. orientalis HKAS127638 Anhui, China
'A. ceciliae' ASIS26247 South Korea
'A. ceciliae' KA12-0758 South Korea
A. sinofulva HKAS92355 Yunnan, China (T)
A. sinofulva EFHAAU207 Anhui, China
A. sinofulva HKAS75058 Yunnan, China
A. sinofulva EFHAAU313 Anhui, China
A. sinofulva EFHAAU118 Anhui, China
A. suborientifulva EFHAAU3559 Anhui, China
A. suborientifulva BZ2013-55 Thailand
A. suborientifulva OR1276 Thailand (T)
A. cf. suborientifulva EFHAAU5291 Anhui, China
A. cf. suborientifulva EFHAAU4437 Anhui, China
'A. orientifulva' STDS-2-10 Japan
'A. orientifulva' KA12-0642 South Korea
A. orientifulva HKAS87937 Yunnan, China
A. orientifulva HKAS32522 Yunnan, China (T)
'A. fulva' ASIS26398 South Korea
A. multicingulata HKAS127631 Anhui, China
A. multicingulata HKAS127630 Anhui, China (T)
A. multicingulata HKAS127634 Anhui, China
A. multicingulata HKAS127632 Anhui, China
A. multicingulata HKAS128054 Anhui, China
A. multicingulata HKAS127634 Anhui, China
A. multicingulata HKAS127635 Anhui, China
A. multicingulata HKAS128049 Anhui, China
A. multicingulata HKAS127637 Anhui, China
A. multicingulata HKAS127636 Anhui, China
A. liquii HKAS36611 Yunnan, China (T)
A. liquii HKAS54568 Yunnan, China
A. liquii HKAS93915 Yunnan, China
A. aff. sinicoflava TRTC156849 Canada
A. aff. sinicoflava TRTC156851 Canada
A. submembranacea MB-001174 Germany
A. constricta BW_Mycoblitz_IV_2 USA
A. changtuia HKAS92100 Heilongjiang, China (T)
A. pachycolea HKAS101422 USA
A. cf. daimonioctantes TRTC155757 Canada
A. umbrinolutea HKAS89201 Sichuan, China
A. umbrinolutea MB-000658 Germany
A. ceciliae C. Bas 9341 The Netherlands
A. fuligineodisca ANDES_F823_NVE324 Colombia
A. fulva N. Arnold2 The Netherlands
A. fulva HKAS96168 Austria
A. tenuifulva HKAS87120 Yunnan, China (T)
A. tenuifulva HKAS58877 Yunnan, China
A. zonata EFHAUU755 Anhui, China
A. zonata HKAS97244 Yunnan, China
A. zonata EFHAUU607 Anhui, China
A. zonata HKAS97240 Yunnan, China (T)
A. zonata EFHAUU709 Anhui, China
A. cf. zonata EFHAAU2254 Anhui, China
A. flavidogrisea BZ2015-44 Thailand (T)
A. luteoparva BZ2015-46 Thailand
A. crebresulcata INPA No. 265178 Brazil
Amanita sp. JEIC0652 Benin
A. cinnamomea BZ2015-45 Thailand (T)
A. cinnamomea BZ2015-48 Thailand
Amanita sp. JEIC0513 Benin
A. craseoderma INPA No. 265158 Brazil
A. cf. sulcatissima TRTC176754 Brazil
A. sulcatissima TRTC176558 Brazil
Amanita sp. JEIC0724 Benin
Amanita sp. JEIC0674 Benin
A. lippiae RET418-2 Brazil (T)
Amanita sp. JEIC0737 Benin
A. subovlispora HKAS128053 Anhui, China
A. subovalispora EFHAAU2621 Anhui, China
A. subovalispora BZ2014-06 Thailand
A. subovalispora BZ2015-70 Thailand (T)
A. olivovaginata SUA138 Pakistan (T)
A. olivovaginata SUA939 Pakistan
A. ovalispora HKAS101406 Fujian, China
A. subovalispora EFHAAU4075 Anhui, China
A. subovalispora HKAS128050 Jiangxi, China
A. subovalispora EFHAAU4480 Anhui, China
A. subovalispora EFHAAU3558 Anhui, China
Amanita sp. JEIC0625 Benin
Amanita sp. JEIC0723 Benin
Amanita sp. JEIC0583 Benin
Amanita sp. INPA No. 265290 Brazil
Amanita sp. INPA No. 265223 Brazil
Amanita sp. TRTC176599 Brazil
Amanita sp. JEIC0599 Togo
Amanita sp. JEIC0510 Guinea
A. neocinctipes HKAS79627 Guangdong, China (T)
A. brunneosquamata BZ2015-73 Thailand (T)
A. olivaceofusca HKAS80243 Fujian, China
A. olivaceofusca HKAS97581 Yunnan, China (T)
A. brunneoprocera EFHAAU4162 Anhui, China
A. brunneoprocera EFHAAU3796 Anhui, China
A. brunneoprocera BZ2015-24 Thailand (T)
A. brunneoprocera HKAS98435 Yunnan, China
A. brunneoprocera HKAS97514 Yunnan, China
A. magnivolvata AF2528 Belgium
A. vladimirii BRNM825829 Czech Republic
A. angustilamellata HKAS24158 Yunnan, China

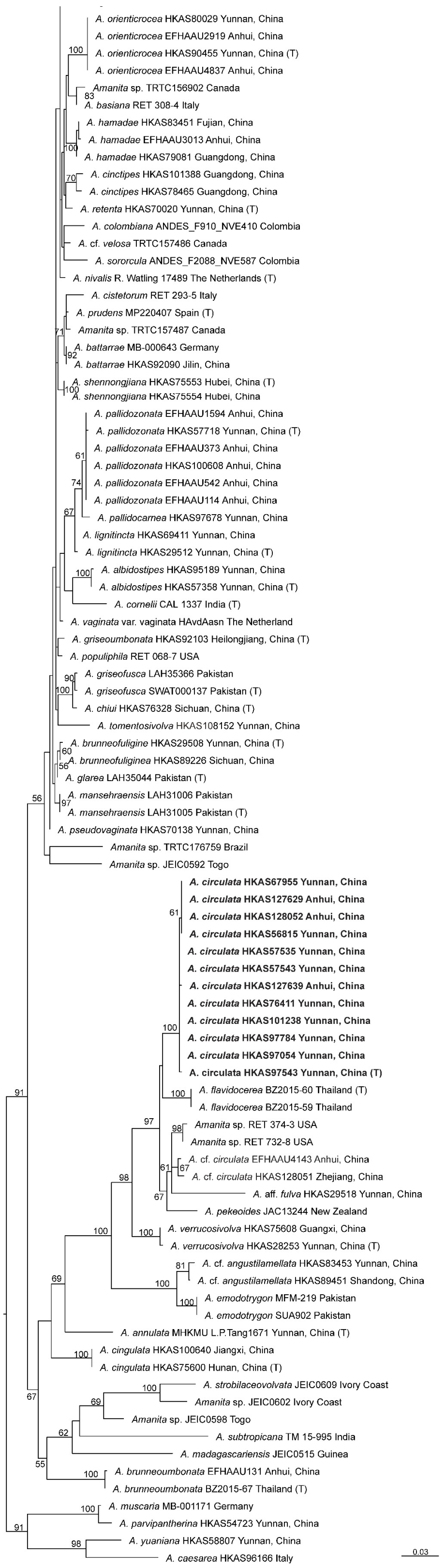

Figure S1. Phylogenetic tree of *Amanita* sect. *Vaginatae* inferred from maximum likelihood analyses based on the nrLSU sequences. Bootstrap values over 50% are shown along the branches. Sequences from type collections are indicated with (T), and new species are in boldface.

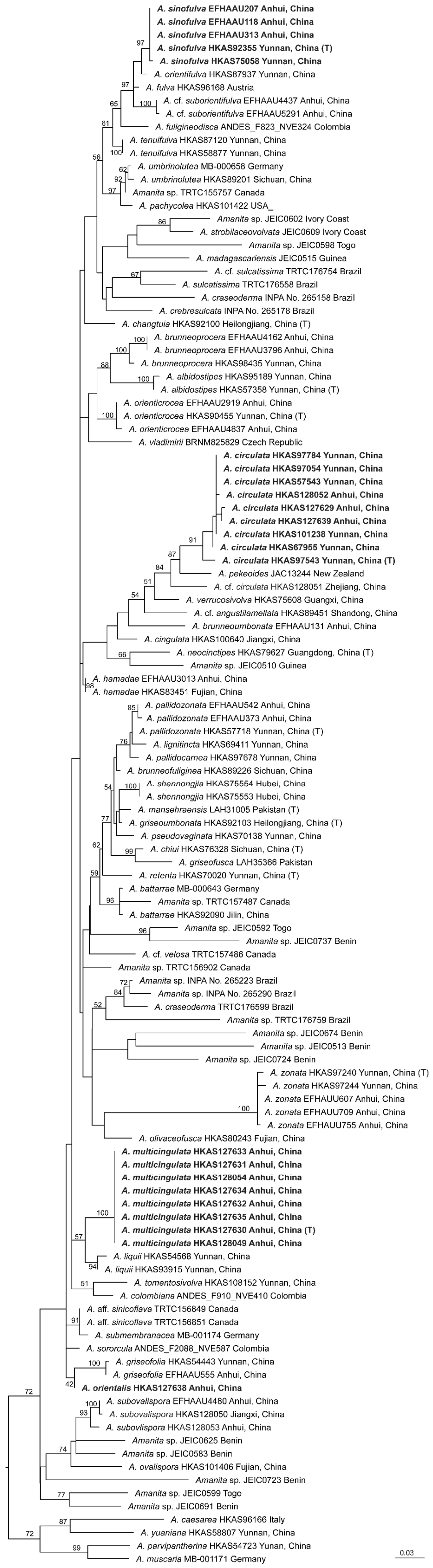

Figure S2. Phylogenetic tree of *Amanita* sect. *Vaginatae* inferred from maximum likelihood analyses based on the *tef1-α* sequences. Bootstrap values over 50% are shown along the branches. Sequences from type collections are indicated with (T), and new species are in boldface.

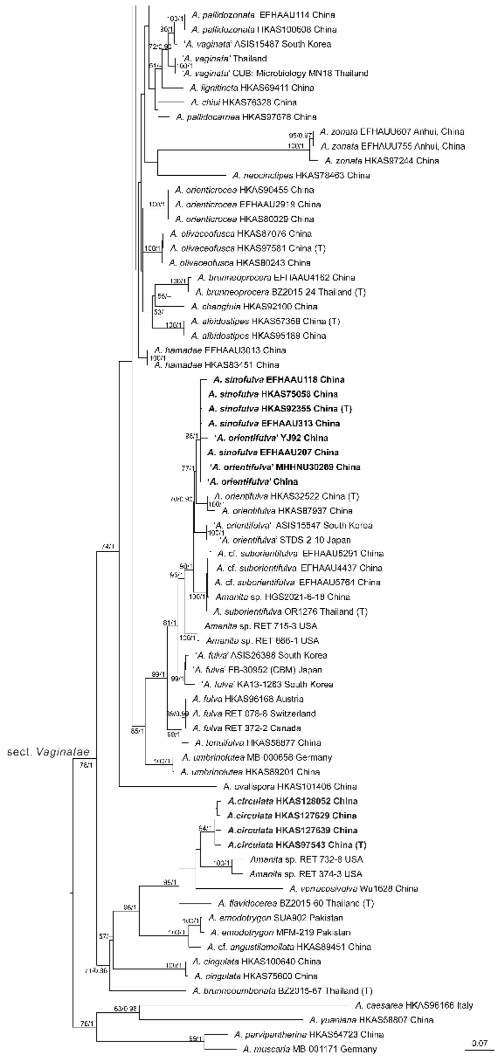

Figure S3. Phylogenetic tree of *Amanita* sect. *Vaginatae* inferred from maximum likelihood analyses based on the *rpb2* sequences. Bootstrap values over 50% are shown along the branches. Sequences from type collections are indicated with (T), and new species are in boldface.

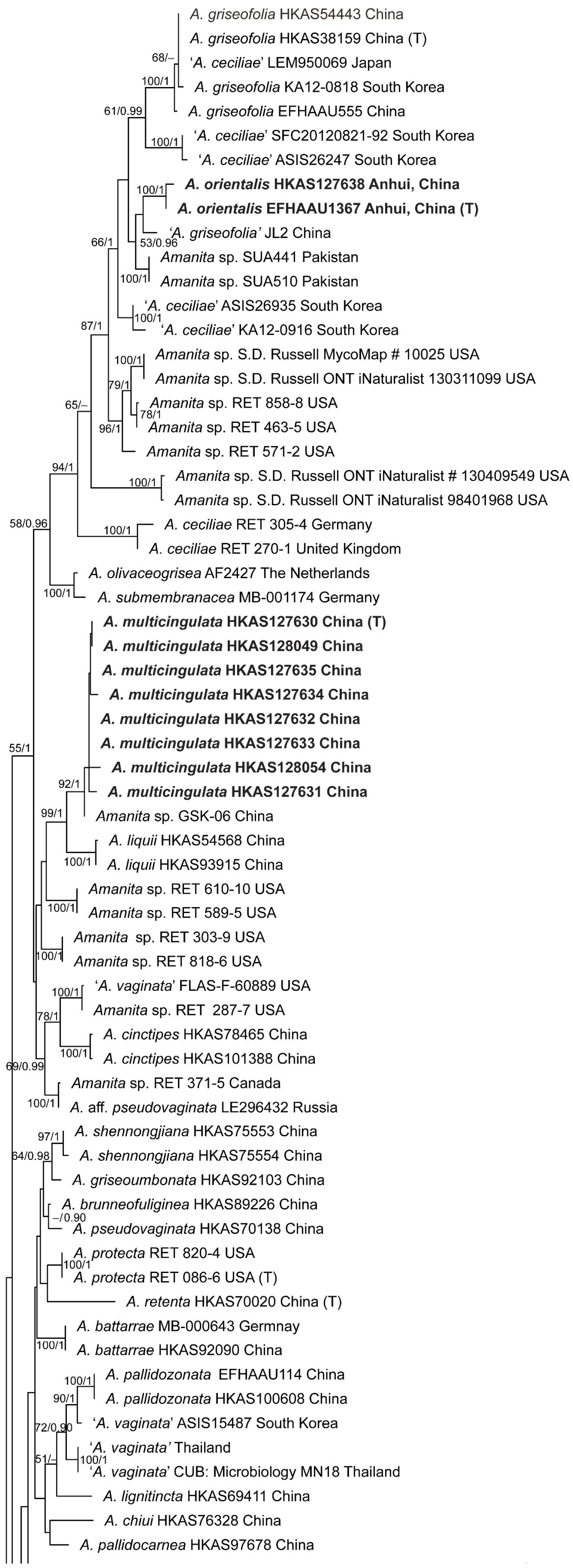
A. griseofolia HKAS54443 China
A. griseofolia HKAS38159 China (T)
'A. ceciliae' LEM950069 Japan
A. griseofolia KA12-0818 South Korea
A. griseofolia EFHAAU555 China
'A. ceciliae' SFC20120821-92 South Korea
'A. ceciliae' ASIS26247 South Korea
A. orientalis HKAS127638 Anhui, China
A. orientalis EFHAAU1367 Anhui, China (T)
'A. griseofolia' JL2 China
Amanita sp. SUA441 Pakistan
Amanita sp. SUA510 Pakistan
'A. ceciliae' ASIS26935 South Korea
'A. ceciliae' KA12-0916 South Korea
Amanita sp. S.D. Russell MycoMap # 10025 USA
Amanita sp. S.D. Russell ONT iNaturalist 130311099 USA
Amanita sp. RET 858-8 USA
Amanita sp. RET 463-5 USA
Amanita sp. RET 571-2 USA
Amanita sp. S.D. Russell ONT iNaturalist # 130409549 USA
Amanita sp. S.D. Russell ONT iNaturalist 98401968 USA
A. ceciliae RET 305-4 Germany
A. ceciliae RET 270-1 United Kingdom
A. olivaceogrisea AF2427 The Netherlands
A. submembranacea MB-001174 Germany
A. multicingulata HKAS127630 China (T)
A. multicingulata HKAS128049 China
A. multicingulata HKAS127635 China
A. multicingulata HKAS127634 China
A. multicingulata HKAS127632 China
A. multicingulata HKAS127633 China
A. multicingulata HKAS128054 China
A. multicingulata HKAS127631 China
Amanita sp. GSK-06 China
A. liquii HKAS54568 China
A. liquii HKAS93915 China
Amanita sp. RET 610-10 USA
Amanita sp. RET 589-5 USA
Amanita sp. RET 303-9 USA
Amanita sp. RET 818-6 USA
'A. vaginata' FLAS-F-60889 USA
Amanita sp. RET 287-7 USA
A. cinctipes HKAS78465 China
A. cinctipes HKAS101388 China
Amanita sp. RET 371-5 Canada
A. aff. pseudovaginata LE296432 Russia
A. shennongjiana HKAS75553 China
A. shennongjiana HKAS75554 China
A. griseoumbonata HKAS92103 China
A. brunneofuliginea HKAS89226 China
A. pseudovaginata HKAS70138 China
A. protecta RET 820-4 USA
A. protecta RET 086-6 USA (T)
A. retenta HKAS70020 China (T)
A. battarrae MB-000643 Germnay
A. battarrae HKAS92090 China
A. pallidozonata EFHAAU114 China
A. pallidozonata HKAS100608 China
'A. vaginata' ASIS15487 South Korea
'A. vaginata' Thailand
'A. vaginata' CUB: Microbiology MN18 Thailand
A. lignitincta HKAS69411 China
A. chiui HKAS76328 China
A. pallidocarnea HKAS97678 China
68/–
100/1
61/0.99
100/1
100/1
53/0.96
66/1
100/1
100/1
87/1
100/1
79/1
78/1
96/1
65/–
100/1
94/1
58/0.96
100/1
100/1
55/1
92/1
99/1
100/1
100/1
100/1
100/1
78/1
100/1
69/0.99
100/1
97/1
64/0.98
–/0.90
100/1
100/1
100/1
90/1
72/0.90
51/–
100/1

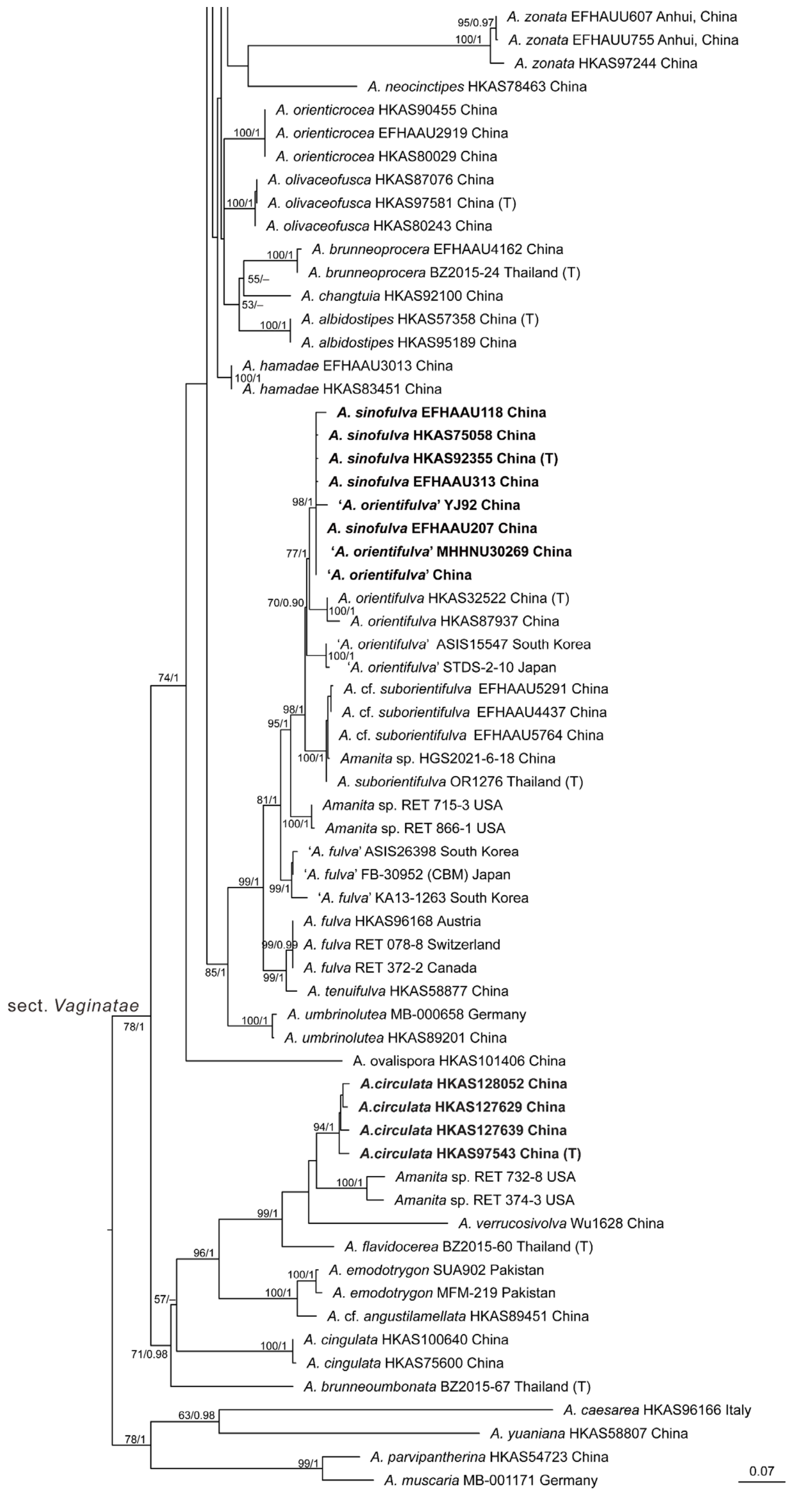

Figure S4. Phylogenetic tree of *Amanita* sect. *Vaginatae* inferred from maximum likelihood analyses based on the ITS sequences. Bootstrap values over 50% and Bayesian posterior probabilities over 0.90 are shown along the branches. Sequences from type collections are indicated with (T), and new species are in boldface.
