# Supplementary material for: Species Diversity of Amanita Section Vaginatae in Eastern China, with a Description of Four New Species
Source: J Fungi (Basel). 2023 Aug 19;9(8):862. doi: 10.3390/jof9080862 (PMC10456051; doi:10.3390/jof9080862)
Supplement: Supplementary file 1 [file jof-09-00862-s001.zip › Table S1.pdf]

Table S1. Voucher information and GenBank accession numbers of the samples used in the phylogenetic analyses of ITS sequences. Sequences newly generated in this study are indicated in bold.

Species	Voucher	GenBank No.	Locality
A. albidostipes	HKAS57358	MH508500	China
A. albidostipes	HKAS95189	MH508501	China
A. cf. *angustilamellata*	HKAS89451	MH508292	China
A. battarrae	HKAS92090	MH508266	China
A. battarrae	MB-000643	MH508267	China
A. brunneofuliginea	HKAS89226	MH508269	China
A. brunneoprocera	BZ2015-24	MF461573	Thailand
A. brunneoprocera	**EFHAAU4162**	**OR042746**	**China**
A. brunneoumbonata	BZ2015-67	MF461579	Thailand
'A. ceciliae'	ASIS26247	KU139510	South Korea
'A. ceciliae'	ASIS26935	KU139512	South Korea
'A. ceciliae'	KA12-0916	KF017930	South Korea
'A. ceciliae'	LEM950069	AB015694	Japan
A. ceciliae	RET 270-1	OK299148	United Kingdom
A. ceciliae	RET 305-4	OK299150	Germany
A. ceciliae	SFC20120821-92	KJ609157	South Korea
A. changtuia	HKAS92100	MH508299	China
A. chiui	HKAS76328	MH508303	China
A. cinctipes	HKAS101388	MH508304	China
A. cinctipes	HKAS78465	MH508305	China
A. cingulata	HKAS100640	MH508309	China
A. cingulata	HKAS75600	KY949582	China
A. circulata	**HKAS127629**	**OR042763**	**China**
A. circulata	**HKAS127639**	**OR042766**	**China**
A. circulata	**HKAS128052**	**OR042764**	**China**
A. circulata	**HKAS97543**	**OR042765**	**China**
A. emodotrygon	MFM-219	MF489730	Pakistan
A. emodotrygon	SUA902	MF489728	Pakistan
A. flavidocerea	BZ2015-60	MF461578	Thailand
'A. fulva'	ASIS26398	KU139519	South Korea
'A. fulva'	FB-30952(CBM)	AB015692	Japan
A. fulva	HKAS96168	MH508371	Austria
A. fulva	RET 078-8	MK141039	Switzerland
A. fulva	RET 372-2	MK522022	Canada
'A. fulva'	KA13-1263	KR673720	South Korea
A. griseofolia	HKAS38159	NR_119498	China
A. griseofolia	HKAS54443	MH508376	China
A. griseofolia	JL2	ON059312	China
A. griseofolia	KA12-0818	KF017934	South Korea
A. griseofolia	**EFHAAU555**	**OR042758**	**China**

A. griseoumbonata	HKAS92103	MH508389	China
A. hamadae	HKAS83451	MH508395	China
A. hamadae	**EFHAAU3013**	**OR042748**	**China**
A. lignitincta	HKAS69411	MH508424	China
A. liquii	HKAS54568	KU714559	China
A. liquii	HKAS93915	MH508427	China
A. multicingulata	**HKAS127630**	**OR042750**	**China**
A. multicingulata	**HKAS127631**	**OR042752**	**China**
A. multicingulata	**HKAS127632**	**OR042754**	**China**
A. multicingulata	**HKAS127633**	**OR042753**	**China**
A. multicingulata	**HKAS127634**	**OR042757**	**China**
A. multicingulata	**HKAS127635**	**OR042755**	**China**
A. multicingulata	**HKAS128049**	**OR042751**	**China**
A. multicingulata	**HKAS128054**	**OR042756**	**China**
A. neocinctipes	HKAS78463	MH508443	China
A. olivaceofusca	HKAS97581	MH508457	China
A. olivaceofusca	HKAS80243	MH508455	China
A. olivaceofusca	HKAS87076	MH508456	China
A. olivaceogrisea	AF2427	MF461569	The Netherlands
A. orientalis	**EFHAAU1367**	**OR042759**	**China**
A. orientalis	**HKAS127638**	**OR042760**	**China**
A. orienticrocea	HKAS80029	MH508464	China
A. orienticrocea	HKAS90455	MH508465	China
A. orienticrocea	**EFHAAU2919**	**OR042747**	**China**
A. orientifulva'	ASIS15547	KP004956	South Korea
A. orientifulva	HKAS32522	NR_137544	China
A. orientifulva	HKAS87937	MH508468	China
A. orientifulva'	MHHNU30269	MK239233	China
A. orientifulva'	STDS-2-10	LC098745	Japan
A. orientifulva'	YJ92	OM867743	China
A. orientifulva'	-	FJ375333	China
A. ovalispora	HKAS101406	MH508478	China
A. pallidocarnea	HKAS97678	MH508482	China
A. pallidozonata	HKAS100608	MH508486	China
A. pallidozonata	**EFHAAU114**	**OR042749**	**China**
A. protecta	RET 086-6	NR_154690	USA
A. protecta	RET 820-4	MT073009	USA
A. pseudovaginata	HKAS70138	MH508531	China
A. aff. *pseudovaginata*	LE 296432	KM658286	Russia
A. retenta	HKAS70020	MH508543	China
A. shennongjiana	HKAS75553	MH508590	China
A. shennongjiana	HKAS75554	MH508591	China
A. sinofulva	**EFHAAU207**	**OR042739**	**China**
A. sinofulva	**EFHAAU313**	**OR042742**	**China**

A. sinofulva	**EFHAAU118**	**OR042740**	**China**
A. sinofulva	**HKAS75058**	**OR042743**	**China**
A. sinofulva	**HKAS92355**	**OR042741**	**China**
Amanita sp.	GSK-06	OK584416	China
Amanita sp.	RET 732-8	MT013999	USA
Amanita sp.	HGS2021-6-18	OK643806	China
Amanita sp.	RET 371-5	MN650861	Canada
Amanita sp.	SUA441	MW425327	Pakistan
Amanita sp.	SUA510	MW425329	Pakistan
Amanita sp.	RET 571-2	MZ385703	USA
Amanita sp.	RET 715-3	MN727380	USA
Amanita sp.	RET 866-1	MT013994	USA
Amanita sp.	RET 463-5	MH753075	USA
Amanita sp.	RET 858-8	MZ318066	USA
Amanita sp.	S.D. Russell MycoMap # 10025	MZ668154	USA
Amanita sp.	S.D. Russell ONT iNaturalist 130311099	OP749510	USA
Amanita sp.	S.D. Russell ONT iNaturalist 98401968	OP549077	USA
Amanita sp.	S.D. Russell ONT iNaturalist # 130409549	OP643044	USA
Amanita sp.	RET 589-5	KY549354	USA
Amanita sp.	RET 610-10	MG992501	USA
Amanita sp.	RET 287-7	OK285686	USA
Amanita sp.	RET 374-3	MN614413	USA
Amanita sp.	RET 303-9	MW592823	USA
Amanita sp.	RET 818-6	MN614382	USA
A. submembranacea	MB -001174	MH508626	Germany
A. suborientifulva	OR1276	MF461584	Thailand
**A. cf. *suborientifulva***	**EFHAAU4437**	**OR042744**	**China**
**A. cf. *suborientifulva***	**EFHAAU5291**	**OR042745**	**China**
A. tenuifulva	HKAS58877	MH508635	China
A. umbrinolutea	HKAS89201	MH508637	China
A. umbrinolutea	MB-000658	MH508641	Germnay
'A. vaginata'	ASIS15487	KP004955	South Korea
'A. vaginata'	CUB: Microbiology MN18	AB458889	Thailand
'A. vaginata'	FLAS-F-60889	MH016871	USA
'A. vaginata'	-	AB451971	Thailand
A. verrucosivolva	Wu1628	KY886480	China
A. zonata	**EFHAAU607**	**OR042761**	**China**
A. zonata	**EFHAAU755**	**OR042762**	**China**
A. zonata	HKAS97244	MH508656	China
**Outgroup**			
A. caesarea	HKAS96166	MH508283	Italy

A. muscaria	MB-001171	MH508442	Germnay
A. parvipantherina	HKAS54723	MH508495	China
A. yuaniana	HKAS58807	MH508653	China
